# Building geochemically based quantitative analogies from soil classification systems using different compositional datasets

**DOI:** 10.1371/journal.pone.0212214

**Published:** 2019-02-19

**Authors:** Mark A. Chappell, Jennifer M. Seiter, Haley M. West, Brian D. Durham, Beth E. Porter, Cynthia L. Price

**Affiliations:** Environmental Laboratory, U.S. Army Engineer Research & Development Center, Vicksburg, Mississippi, United States of America; The University of Sydney, AUSTRALIA

## Abstract

Soil heterogeneity is a major contributor to the uncertainty in near-surface biogeochemical modeling. We sought to overcome this limitation by exploring the development of a new classification analogy concept for transcribing the largely qualitative criteria in the pedomorphologically based, soil taxonomic classification systems to quantitative physicochemical descriptions. We collected soil horizons classified under the Alfisols taxonomic Order in the U.S. National Resource Conservation Service (NRCS) soil classification system and quantified their properties via physical and chemical characterizations. Using multivariate statistical modeling modified for compositional data analysis (CoDA), we developed quantitative analogies by partitioning the characterization data up into three different compositions: Water-extracted (WE), Mehlich-III extracted (ME), and particle-size distribution (PSD) compositions. Afterwards, statistical tests were performed to determine the level of discrimination at different taxonomic and location-specific designations. The analogies showed different abilities to discriminate among the samples. Overall, analogies made up from the WE composition more accurately classified the samples than the other compositions, particularly at the Great Group and thermal regime designations. This work points to the potential to quantitatively discriminate taxonomically different soil types characterized by varying compositional datasets.

## Introduction

Developing models for predicting soil behavior, such as biogeochemical reactions or the environmental fate of contaminants, typically begins with the application of generalized mass balance models that incorporate hydrologic and physical processes driving transport with experimentally determined parameters describing (i) the batch equilibrium distribution of the solutes between the soil and water phases (i.e., sorption distribution coefficient or K_D_) and (ii) kinetic parameters describing nutrient or solute persistence, such as via abiotic/biotic degradation. Attempting to develop data that is broadly applicable to a wide variety of environmental conditions, experimental soil systems are generally manipulated to show simple connections between particular soil characteristics and its associated soil “behavior”. For example, the environmental fate of contaminants is often considered in terms of comparing the contaminant’s abiotic sorption on soils across varying soil organic carbon, clay, or metal oxide concentrations. For charged solutes, these systems are typically further manipulated to study the impacts of pH or redox conditions through the addition of acids or bases, and organic carbon. When studying biotically mediated contaminant fate, the experimental systems are even further manipulated to hold soil moisture, aeration, and temperature constant. While these experimental conditions are useful for elucidating potential mechanistic information related to how a soil’s properties influence the eventual fate of contaminants, it is reasonable to assume that these manipulations may also promote substantial deviations in the natural biogeochemical behaviors. Thus, there is a risk that developing soil behavioral information using highly idealized systems could ultimately frustrate our ability to predict the environmental fate for natural soil types.

Much of the error in biogeochemical modeling originates from uncertainties in the parameters describing the soil and sediment compartments [1, 2]. Attempts to overcome this problem procedurally through standardized experimental or analytical methods (such as via published ASTM, USEPA, or OECD methods) are challenged by our current inability to define thermodynamically the initial state of a soil system, as can be done in systems with homogenous solids–a challenge largely occurring due to the inherent heterogeneity of soils. Inherent soil heterogeneity makes it theoretically difficult to represent any particular soil biogeochemical behavior with a single “universal” coefficient, such as a solute partitioning coefficient (K_D_) for the equilibrium contaminant sorption or a 1^st^-order kinetic coefficient for indicating a contaminant’s persistence in soil. In spite of these theoretical and practical limitations, these types of environmental fate parameters are almost universally employed in biogeochemical models. While the uncertainty in the models is nearly impossible to quantify, these models remain popular given that the environmental science community generally lacks any good alternatives. Some models attempt to reduce the uncertainty in using single universal coefficients to describe all contaminant interactions by utilizing different fate parameters for different environmental phases–a modeling approach employed in popular multicompartment models [3]. A second alternative approach may involve substituting single fate coefficients with empirical functions based on univariate soil physical and chemical characteristics [4, 5]. Here, it is assumed that one can better tune an environmental model to a particular site of interest by incorporating the properties of soil. The dilemma here is that the analogy (defined in this paper as the “structural” construct underlying data interpretation) for a site of interest remains ambiguous and highly uncertain. This fact occurs because the analogy was built solely on quantitative characteristics without any consideration of the soil’s “context”. Without defining the soil’s context or class, it is impossible to fully appreciate the importance of the quantitative characteristics, and ultimately, discriminate one soil from another [6]. Lacking the ability to unambiguously discriminate one soil from another makes it difficult to justify extrapolating any prediction of a soil’s biogeochemical behavior generated from a poorly defined analogy to other sites of interest. Thus, a (perpetually) repeating, time-consuming, and expensive pattern that has emerged in the environmental science community requiring that every new site of interest conduct a corresponding new round of investigations.

Stepping back from the approaches discussed above to consider the pedomorphological features as primary descriptors of soil in environmental modeling provides some helpful perspectives. The most common pedomorphologically based soil taxonomic systems are the NRCS system and the Food and Agricultural Organization of the United Nations Educational, Scientific, and Cultural Organization (FAO-UNESCO) system—with its emerging update as the World Reference Base or WRB [7, 8]. These systems were designed to provide a common criteria and terminology for not only distinguishing different soil types, but also discussing soil behavior and application, such as in agriculture or other technological uses of soils [9]. While pedomorphological considerations readily discriminate among different soil types, these distinctions are largely qualitative (based on visual inspection), supplemented by limited quantitative characterization data. Thus, the corresponding analogies explaining the similarity among different soil types in these classification systems are largely qualitative, containing quantitative information that is insufficient to develop a distinctive signature of each soil type. In theory, building quantitative (or numeric) analogies would make the highly discriminating capability of soil classification systems accessible to quantitative modeling, such as predicting soil biogeochemical behavior relative to a designated soil type. To be clear, soil taxonomic systems excel at providing the necessary “context” by which soil properties may be interpreted.

As formulated materials, Norris [10] considered soils as “multivariate entities”, arguing that a more accurate description of a soil is made by considering multiple soil characteristic variables than any single variable alone. Numerous studies over the past few decades have emphasized this idea (to one degree or another) for soils and other environmentally relevant solids and matrices. Examples include identifying terpane chemical signatures based on different source geologies [11]; discriminating soil geochemical baseline for metal contaminated sites [12]; chemically distinguishing coal fly-ash from background sediment after a major spill in the U.S. [13]; discriminating minerals collected by NASA’s Mars Science Laboratory rover based on remote laser-induced breakdown spectroscopy [14]; distinguishing rainwater by age [15] or simply chemically distinguishing sediments based on collection site [16, 17] and time of deposition relative to establishment of the U.S. EPA’s Clean Water Act [18]. Not only can these approaches be useful for discriminating characterization data, but also discriminating environmentally mediated processes. For example, multivariate models have been generated for correlating soil fungal and bacterial communities [19–21], and organic and inorganic contaminant sorption distribution coefficients (K_D_) [22, 23] and degradation [24, 25] to soil “types” or classes. In these examples, soil type was not reported in terms of organized pedomorphic taxonomic systems, but generalized in terms of the geographical and vegetative criteria, such as desert soils, forest soils, coniferous soils, hard-wood growth forests, etc.

Aitchinson’s landmark revelations regarding the nature of compositional data are extremely useful for circumventing latent structural artifacts in characterization data. The theoretical basis for compositional data has been reviewed elsewhere [26]. However, it is important to point out that nearly all soil characterization data is inherently compositional in nature, and thus, CoDA theoretical considerations are not only appropriate but necessary. Consider that nearly all quantitative soil characterization data are reported in units of concentration, such as part per million (e.g., mg kg^-1^) or as a percentage, and composed of all positive (non-negative) values. As such, these traits are key indicators of compositional data, implying that if all parts of the composition were analyzed, they would sum to 100%. This means that the composition is “closed”; a “fixed-closure bias” is evident in the structure of compositional data that appears distorted in Euclidean space. These distortions give way to potentially spurious statistical correlations obtained using classical structural methods. Thus, analysis must be conducted either in “compositional” space, or in real space after the data is correctly transformed.

This paper describes our efforts to develop quantitative analogical models for different soil types, utilizing taxonomic descriptors originating from the NRCS soil classification system. Here, the analogies were built using soil characterization data partitioned into three different compositions described hereafter.

## Methods

### Soil selection, collection, processing, and characterization

**Fig 1:**
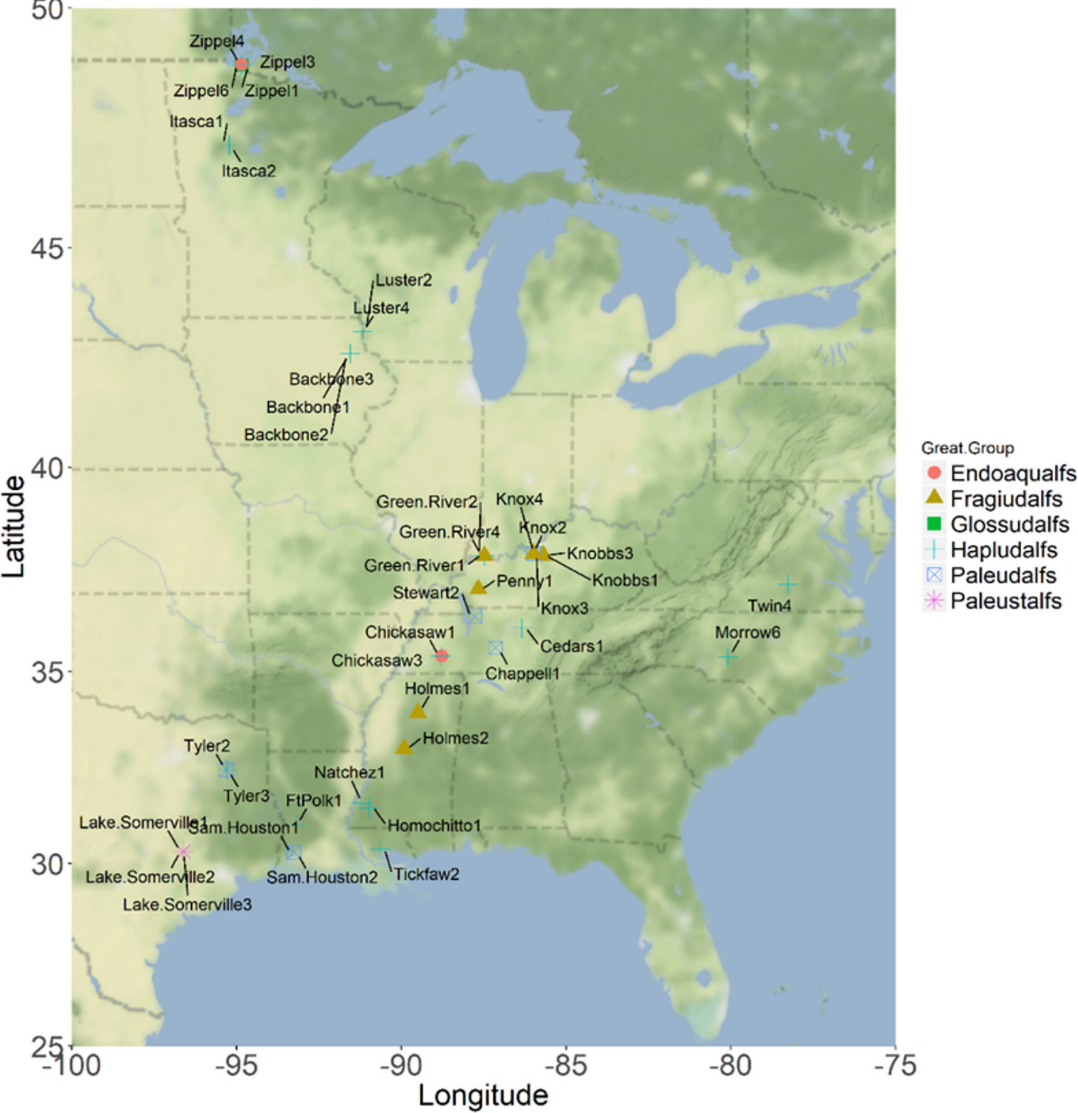
Map showing location of Alfisols collected throughout the Eastern and Midwestern U.S. Location markers are colored with respective to the soils’ NRCS Great Group taxonomic designations. The map was created using the ggmap package [27] for R.

Pre-selected soil series were targeted based on interest in populating different subcategories of soil types within the Alfisols order. We preferentially sought out pristine, non-anthropogenically disturbed sampling sites containing native vegetation. For this reason, we targeted our collections at State Parks (after receiving both verbal and written permissions from Park Rangers) and historically non-used areas (as recommended by range managers) on U.S. Army bases in the Eastern and Midwestern U.S. At each site (Fig 1 and Table 1) the profile was sampled using a soil corer to determine if the NRCS-designated soil series descriptions qualitatively matched the observed features. Once satisfied, we collected samples by excavating soil approx. 10–20 inches (25.4–50.8 cm) down the profile, sampling each horizon based on visible delineators and the soil series descriptions. Samples were collected in plastic bags, sealed, and shipped to our laboratory in Vicksburg, MS, USA, where they were air-dried, sieved, and ground using a soil grinder to pass through a 2-mm sieve, homogenized using an acoustic mixer, and then stored in polypropylene bottles at 4°C until used. The soil mixture was subsampled in triplicate, and geochemically characterized using common soil characterization methods (S1 Table) used in environmental science for focusing on surface interfacial properties.

**Table 1:** Samples collected including soil type and geographical coordinates of the location.

Soil	Suborder	Great Group	Soil series	Latitude	Longitude
**Backbone1**	Udalfs	Hapludalfs	Basset	42.64347	-91.5553
**Backbone2**	Udalfs	Hapludalfs	Whalan	42.64392	-91.5553
**Backbone3**	Udalfs	Hapludalfs	Backbone	42.64402	-91.5482
**Cedars1**	Udalfs	Hapludalfs	Talbott	36.09443	-86.3402
**Chappell1**	Udalfs	Paleudalfs	Braxton	35.5943	-87.1452
**Chickasaw1**	Aqualfs	Endoaqualfs	Tooterville	35.38949	-88.7779
**Chickasaw3**	Udalfs	Hapludalfs	Lexington	35.3863	-88.8016
**Fort Knox1**	Udalfs	Fragiudalfs	Bedford	37.93652	-85.9843
**Fort Knox2**	Udalfs	Paleudalfs	Vertrees	37.93482	-85.9767
**Fort Knox3**	Udalfs	Paleudalfs	Baxter	37.92655	-85.9905
**Fort Knox4**	Udalfs	Fragiudalfs	Nicholson	37.92591	-85.9914
**Fort Polk1**	Udalfs	Hapludalfs	Eastwood	31.0238	-93.2014
**Green1**	Udalfs	Fragiudalfs	Hosmer	37.8774	-87.4791
**Holmes2**	Udalfs	Fragiudalfs	Loring	33.02993	-89.9118
**Itasca1**	Udalfs	Hapludalfs	Debs	47.19655	-95.2204
**Itasca2**	Udalfs	Hapludalfs	Lengby	47.19265	-95.2133
**Knob1**	Udalfs	Fragiudalfs	Otwell	37.88947	-85.6972
**Knob3**	Udalfs	Fragiudalfs	Lawrence	37.89042	-85.6968
**Lake Sommerville1**	Ustalfs	Paleustalfs	Singleton	30.3185	-96.6148
**Lake Sommerville2**	Ustalfs	Paleustalfs	Rehburg	30.31868	-96.6124
**Lake Sommerville3**	Ustalfs	Paleustalfs	Eufaula	30.31817	-96.6111
**Luster2**	Udalfs	Hapludalfs	Fayette	49.13707	-91.1867
**Luster4**	Udalfs	Hapludalfs	Dubuque	43.13458	-91.1867
**Morrow6**	Udalfs	Hapludalfs	Enon	35.36652	-80.0913
**Natchez1**	Udalfs	Hapludalfs	Memphis	31.59822	-91.2169
**Penny1**	Udalfs	Fragiudalfs	Zanesville	37.06266	-87.6726
**Sam1**	Udalfs	Paleudalfs	Bienville	30.29563	-93.2682
**Sam2**	Udalfs	Paleudalfs	Glenmora	30.30097	-93.2555
**Tickfaw1**	Aqualfs	Albaqualfs	Springfield	30.38292	-90.6475
**Tickfaw2**	Udalfs	Hapludalfs	Colyell	30.38147	-90.6478
**Twin4**	Udalfs	Hapludalfs	Wilkes	37.17608	-78.2762
**Tyler2**	Udalfs	Paleudalfs	Pickton	32.48015	-95.3013
**Tyler3**	Udalfs	Hapludalfs	Redsprings	32.47345	-95.2973
**Zippel1**	Udalfs	Glossudalfs	Suomi	48.84783	-94.8471
**Zippel3**	Udalfs	Hapludalfs	Karlstad	48.85832	-94.8373
**Zippel6**	Udalfs	Hapludalfs	Chilgren	48.84996	-94.8533

### Multivariate modeling for analogy generation

All statistical modeling was conducted using R statistical computing software [28] through the graphical interface RStudio [29]. Soil taxonomic information for the collected soil series were extracted from the USDA-NCSS soil databases using the “soilDB” package [30]. Soil characterizations were conducted using both chemical and physical analysis (see Supporting Information). Soil chemical properties were quantified based on solutions analyzed for as many solutes as possible in order to capture any lingering (and often ignored) covariate information. The characterization data was divided into three different compositions based on the experimental methods employed: (i) water-extracted (WE), representing a composition measurement obtained by washing the soils with a dilute salt solution (containing 20 variables), (ii) Mehlich-III extracted (ME), representing a composition obtained using a weak acid solution (containing 14 variables), and (iii) particle size distribution (PSD, containing 3 variables). The ratio of variables to observations conforms to more “tolerant” rules of the appropriate dimensionality required for stable PCA results [31]. In order to develop the analogy models, all solution characterization data were uniformly converted to homogeneous mass concentration units (mg kg^-1^), including pH using -10^(pH), and electrical conductivity (EC) by assuming 1 mS cm^-1^ = 10 meq(+) L^-1^ = 640 mg L^-1^ total salts [32]. The exception to this conversion was our calculation of cation exchange capacity (CEC), in units of cmol(+) kg^-1^, which was calculated by summing the charge-equivalent Na, Ba, Ca, Mg, and K concentrations measured from the Mehlich-III extractions.

Missing data patterns (due to analytical non-detects) were studied using the “zCompositions” package [33] for R. Missing variables that exceeded 50% of the total samples were removed from the matrix (see Supporting Information). As a result, we removed ortho-PO_4_, B, P, S, Sn, Ti, and Sr solutes from the WE composition, and P, Sr, S, Th, Sn, and Ti from the ME composition. Missing “zeroes” in all compositions were replaced using zComposition’s multivariate imputation method from a matrix containing estimates of the analytical detection levels of the missing characterization data. The final variable set was closed to the unit sum of 100 using the *clo* command in the “compositions” package [34].

Analogies were created by modeling the data using robust principal component analysis (PCA) via *pcaCoDa* command in the “robcompositions” package [35]. Here, PCA was performed first using an isometric log-ratio (ilr) transformation of the dataset (using a default basis set), followed by conversion to a centered logratio (clr) transformed data for interpreting results. The clr transformation is defined as:

$$\mathrm{clr}(x)=\left[\ln\frac{x_{1}}{g(x)},\ldots ,\ln\frac{x_{D}}{g(x)}\right]$$ (1)

Here, the terms in Eq 1 represents the logratio of each variable and the geometric mean across the different components as $g(x)={\left(\prod_{i=1}^{D}x_{i}\right)}^{1/D}$. Given no particular algorithms exist for determining the stopping point of PCA for compositional data, the optimum number of principal components (PCs) was selected based on the recommendations by Jackson [36]. Here, we considered a combination of the calculated eigenvalues and percent explained variance for each component, as well as the overall shape of the Scree plot (See Supporting Information). According to Jackson [36], agreement between the Scree criterion and eigenvalues > 1 is typically exhibited in structured data. Irregular Scree plots are more indicative of random variation within the data; thus, we opted to choose PCs with eigenvalues > 1. Robust clr-loadings were extracted from the PCA and plots were studied for opportunities to remove variables that were redundant, or otherwise, provided no significant information. Afterwards, robust clr-scores were extracted from the model and used for statistically discriminating the samples via linear discriminant analysis (LDA) using the “MASS” package [37]. Shapiro-Wilk tests showed that all clr-transformed data conformed to a normal distribution (see Supporting Information).

## Results

### PCA-based soil analogies for the three different compositional datasets

**Fig 2:**
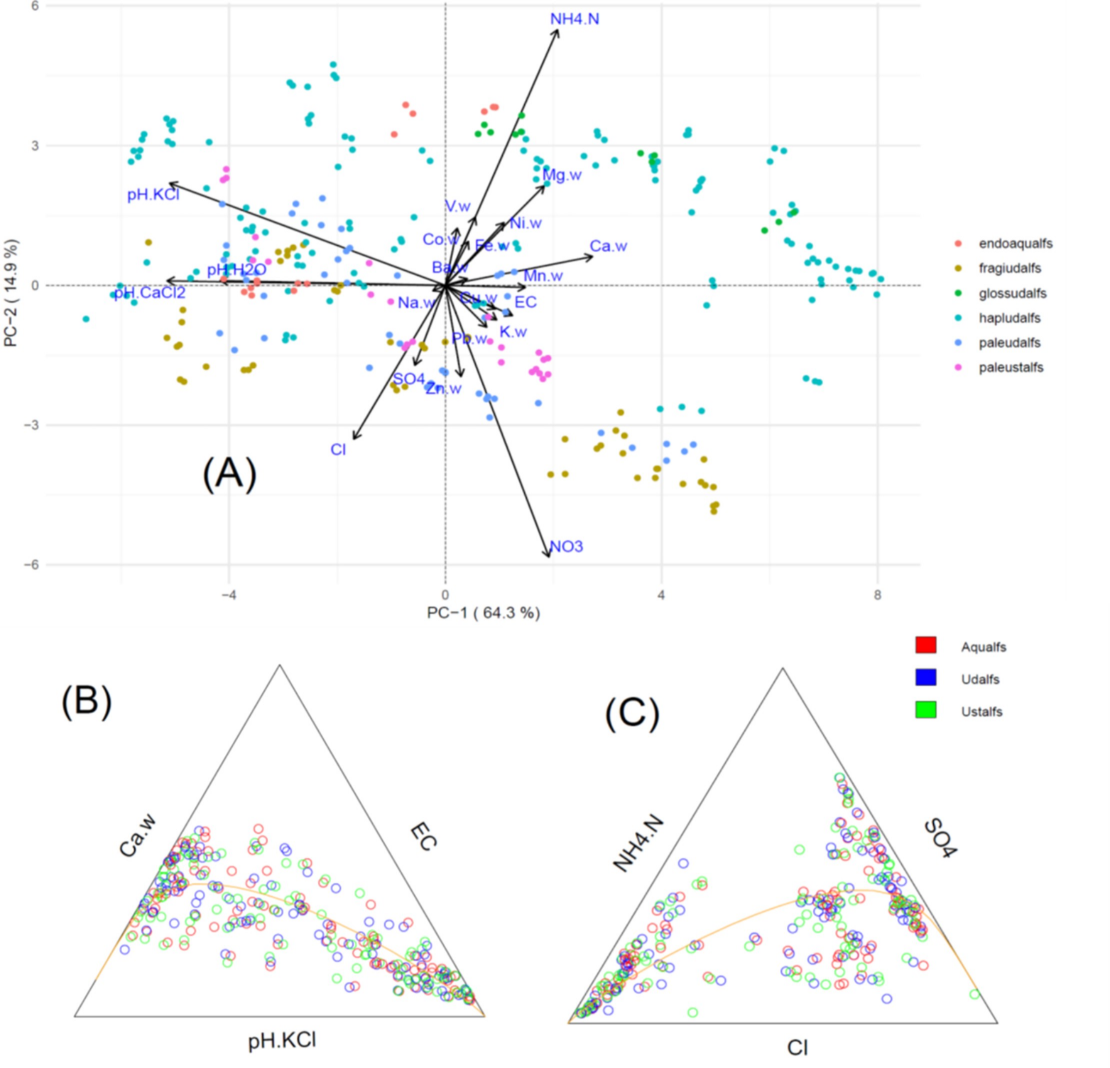
Statistical plots for the water-extracted (WE) composition (suffix “.w” refers to a constituent from the WE composition). (A) Clr-transformed biplots for the full composition with samples grouped based on the Great Group designation. (B) ternary principal component (t-PCA) plot for the pH.KCl-Ca-EC subcomposition, with samples grouped based on the Suborder designation. (C) t-PCA plot for the NH_4_.N-Cl-SO_4_ subcompositions, grouped based on the Suborder designation.

Fig 2 shows the biplots from the PCA, articulating how the variables making up the different compositions correlated with the samples used to define the soil analogies. It is important to recall that statistical biplots are interpreted differently for compositional data. For typical covariance-based biplots, the vector length is a graphical representation of the contribution of each variable to the linear-combination equation making up the model’s explained variance. However, for compositional data, vector lengths graphically represent the deviation of the variable from the model center (i.e., the plot origin in reduced space), or the geometric mean (g(x)) in Eq 1- thus, the clr transformation) of each variable [38]. Compositional biplots are interpreted by studying the log ratios between pairs of variables x_i_ and x_j_ represent as:

$$\log(x_{i}/x_{j})=\log(x_{i})-\log(x_{j}).$$ (2)

Eq 2 is simple but important to keep in mind when considering the relationship between any pair of variables. Large log ratios indicate high variation between the two variables, while the inverse is true for small log ratios. Log ratios close to zero indicate very little difference between the variables, opening the way for possibly combining the variables to filter out non-essential information. Measured link distances (lines drawn from the tip of one variable vector to another) graphically represent the log-ratio variances, while the pairwise vector lengths show their relative standard deviation. These basic properties of the compositional data analysis hold important implications for soil analysis; that soil data collections can be intelligently prioritized based on the most important information. What constitutes the most “important” information for discriminating different soil “types” as well as identifying important variables driving soil reactions remains unclear, however, the fact that any one subcomposition is scale invariant [39] may simplify the choice of characterization variables. All this assumes that highly significant and robust analogies can be developed.

The water-extracted (WE) composition was described with a 5-PC model (based on shape of the Scree plot and eigenvalues > 1) with the PC 1–2 representing 80% of the explained variance, making it a good candidate for exploratory analysis using biplots. In particular, we examined the variance matrix and loading plots to explore logratios and subcompositions of low and high variance (Fig 2A), looking for opportunities to reduce the size of the composition [40]. Log ratios contributing least to the total variance of the PCA model were log(Ba/Na), log(Na/Zn), representing approx. 0.7 and 1.4% of the explained variance, respectively (see Supporting Information for the logratio variation matrix). However, none of these rays for the different log ratios overlapped in the biplot (Fig 2A), therefore, were not appropriate to combine into a single variable. On the other hand, rays for log(V/NH_4_.N), log(Ni/Mg), log(Fe/V), log(Cu/EC), and log(pH.H_2_O/pH.CaCl_2_) noticeably overlapped, but the links were sufficiently large to discourage further combining these variables as well.

After evaluating the WE composition for nonsignificant information, we examined the biplot for evidence of collinearity among the remaining variables [39–41]. Collinear variables were suggested by different rays situated on a common line, whether in the same or opposite direction. The most obvious example in the WE composition was observed in the long link connecting the pH.KCl and Ca variables along the axes of their rays (Fig 2B). Calculations showed that a pH.KCl-Ca-EC subcomposition contributed only 0.045% to the total variance. A ternary principal component (t-PC) analysis (Fig 2B) showed the data points clearly following the line defining the principal axis. The first PC, explaining 95% of the variance in the subcomposition was highly loaded by pH.KCl. The NH_4_.N-SO_4_-Cl subcomposition also appeared similarly collinear, however, considerable scattering of the data around the principal axis (Fig 2C), probably reflecting the large number of nondetects in the Cl data that were modified by the multivariate imputation technique.

In the biplots (Fig 2), clustering was evident for most of the samples (represented as clr-transformed scores), except the populations appeared “stretched” across the principal axis, suggesting the presence of leverage outliers (thus, the use of robust principal component algorithm). This outcome was expected given diversity of taxonomically distinct soil types captured in this work under the Alfisols Order. From the biplot in Fig 2, narrow linear boundaries or margins separating several of the clusters (at the Great Group level) were apparent for PC 1–2, particularly for the Fragiudalfs, Paleudalfs, and Paleustalfs samples, largely occupying negative PC-2 space. On the other hand, the Hapludalfs samples appeared to cluster in two separate populations, one at negative PC-1 and the other in positive PC-1 space, with the small Glossudalfs group.

**Fig 3:**
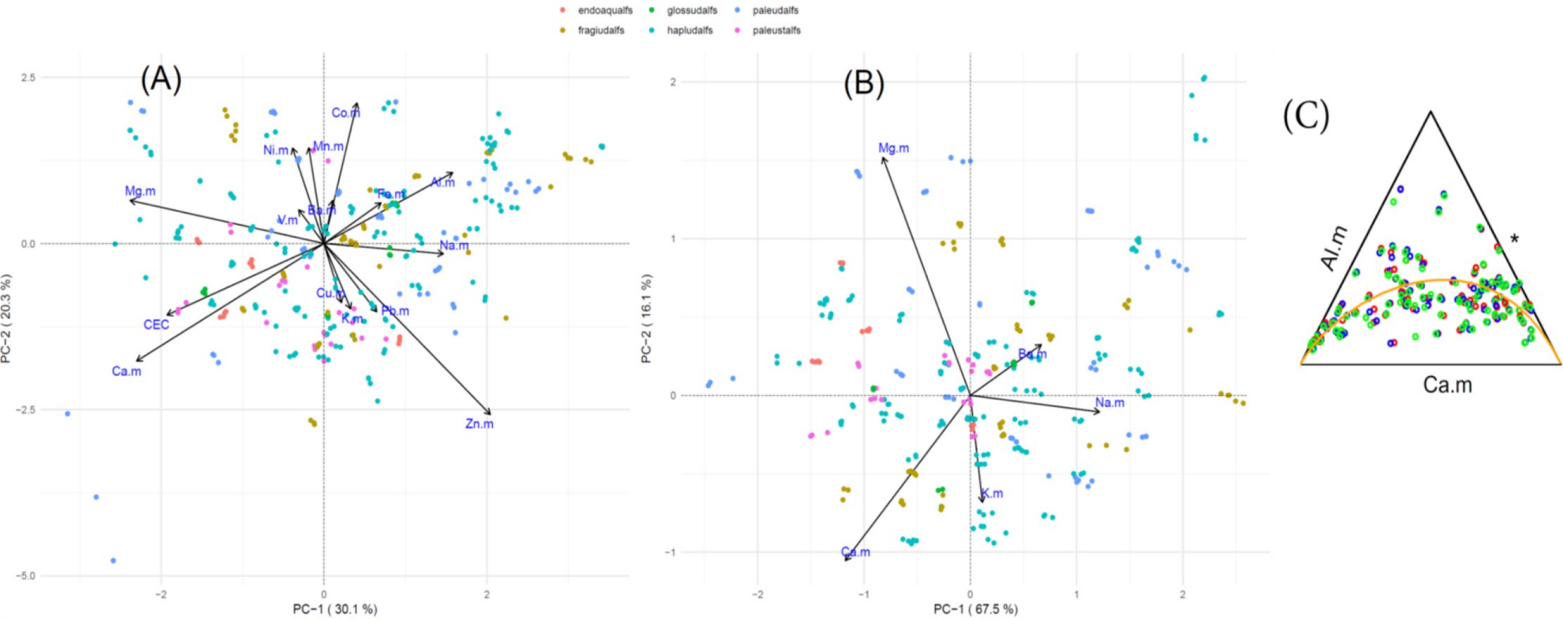
Statistical plots for the ME and CEC compositions (“.m” suffix refers to a constituent of the ME composition). (A) clr-transformed biplots for the ME composition, with samples grouped based on the Great Group designation. (B) clr-transformed biplots for the CEC composition, with samples grouped based on the Great Group designation. (C) Results of a marginal analysis of the CEC composition, where third component, “*”, represents the geometric mean of the Al and Ca parts, and the orange line representing the first principal component.

For the ME composition, we obtained a two PC model (based on the eigenvalues > 1 given the unusual shape of the Scree plot), explaining only 50% of the total variance. Sample clustering in the biplot based on the Great Group designation was much less apparent with the ME composition, thus we expected this composition to be less discriminating than the WE composition. Furthermore, there were limited opportunities to simplify the composition. For example, the very low variance of log(Ca/CEC) suggested that the two variables may be redundant, however, the two rays did not overlap in the biplot (Fig 3). This lack of overlap may be an artifact of the low explained variance of the PCA model, with the low variance of the log(Ca/CEC) possibly suggesting the links were either very small or orthogonal [39]. Whichever the case, we decided against combining variables in the ME composition given the low explained variance of the overall PCA model. Also, t-PCA analysis did not find any strong collinear relationships among the reduced ME composition (plots not shown).

We realized that the ME composition in itself contained a unique subcomposition describing the exchangeable cation content at the soil surface. Thus, we created a separate CEC subcomposition from the exchangeable cations, Na, Ba, Ca, Mg, and K, and added exchangeable Al given the appearance of collinearity in Fig 2B (which could not be confirmed because of the low explained variance of the model for the ME composition). From the CEC subcomposition, we selected a 3-PC model that explained 84% of the total variance in the data, representing a substantial improvement over the full ME composition. Interestingly, the biplot (Fig 3B) was similar to that obtained in Fig 3A, suggesting that the variance among the exchangeable cation logratios dominated the structure in the ME composition (i.e., subcompositional coherence). The log (Ca/Mg) represented the largest link, suggesting that the log(Ca/CEC) in the ME composition was probably redundant. With the higher explained variance of the CEC subcomposition, the collinear relationship between Al and Ca was more evident (Fig 3C).

**Fig 4:**
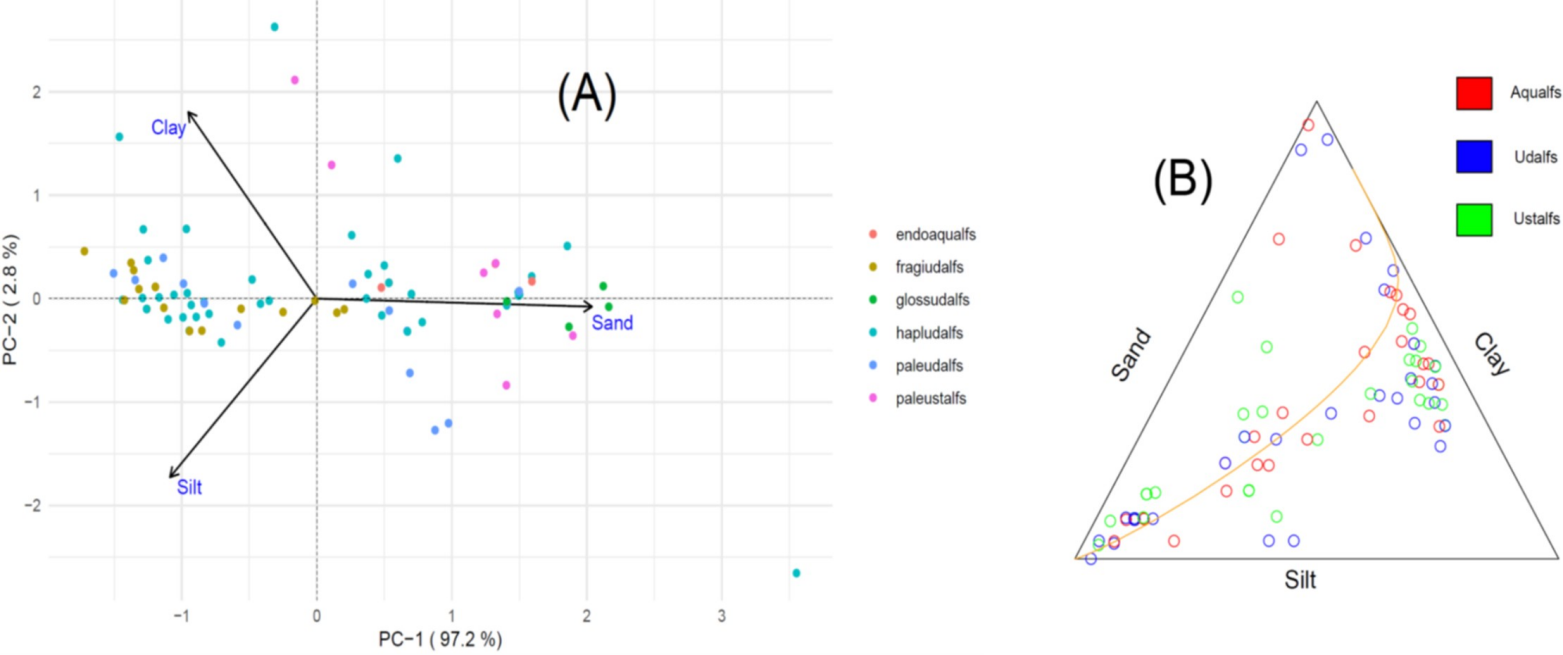
Statistical plots for the PSD composition grouped according to Great Group designation. (A) Clr-transformed biplot for the full PSD composition. (B) t-PCA plot for the full PSD composition, with the orange line in the t-PCA plot representing the principal axis.

The PCA model for the PSD composition was limited to two PCs, given that the composition consisted of only three parts. Thus, we plotted this PCA results as both in compositional and clr-transformed biplots (Fig 4). The first PC explained 98% of the total variance, and highly loaded by %Sand. It was more difficult to observe clustering in this data, in part due to the smaller populations (as described previously), however, there were some apparent relationships. For example, the Fragiudalfs appeared to cluster predominantly in negative PC-1, within the log(Clay/Silt) (Fig 4A), while the “older” Paleudalfs and Paleustalfs mapped out in positive PC-1, better described by the log(x/Sand). This clustering is somewhat evident in the t-PCA plot as well (Fig 4B), with clustering at higher %clay, and toward the %Silt vertex.

LDA tests were conducted to determine the extent in which the different classes were discriminated based on their PCA models. Here, the cross-validated results were used as the test set. Overall, the WE composition (Table 2) was highly discriminating for most of the classes–an outcome implied by the linearly separable clustering apparent in the biplot (Fig 2). The WE composition best discriminated the general Suborder class, with most misclassifications occurring due to the model confusing samples as Udalfs (representing the largest population of samples). However, at the Great Group level (representing one level down in the NRCS taxonomy), the WE composition was much less accurate. While Hapludalfs and Fragiudalfs were correctly classified 73 and 80% of the time, the other Great Groups were confused by the Hapludalfs designation. Similarly, the WE composition most consistently identified the correct temperature classes (under the Family name), with the accuracy for all three thermal regimes ranging from 87–91%. The mesic and thermal regimes were the most confused with each other by the WE composition, while no thermic regime samples were confused with the frigid temperature class.

**Table 2:** Misclassification matrix from linear discriminant analysis testing of the accuracy of PCA (containing 5-PCs) model to predict the membership of the Alfisols based on the water-extracted (WE) compositional data. Percent correct classifications are shown on the diagonal (in bold) while percent misclassification samples are shown off-diagonal.

Designation	Class	Predicted class						Total accuracy
Suborder		Aqualfs	Udalfs	Ustalfs				
	Aqualfs	**0(0)**	100					
	Udalfs		**98**	1				
	Ustalfs		33	**67**				91
Great Group		Endoaqualfs	Fragiudalfs	Glossudalfs	Hapludalfs	Paleudalfs	Paleustalfs	
	Endoaqualfs	**0**	60	7	33			
	Fragiudalfs	2	**80**		18			
	Glossudalfs			**92**	8			
	Hapludalfs	3	5	4	**73**	13	2	
	Paleudalfs	2	50		27	**21**		
	Paleustalfs				8		**92**	64
Horizon		Subsurface	Surface					
	Subsurface	**84**	16					
	Surface	56	**46**					69
Family—mineralogy		kaolinitic	mixed	siliceous	smectitic			
	kaolinitic	**17**	83					
	mixed		**92**	1	7			
	siliceous	5	36	**33**	26			
	smectitic		33	27	**40**			74
Family—clay activity		active	none	semiactive	superactive			
	active	**76**	12	9	3			
	none*	28	**58**		14			
	semiactive	52	14	**33**				
	superactive		9		**91**			69
Family—temperature		frigid	mesic	thermic				
	frigid	**91**	4	4				
	mesic		**87**	13				
	thermic		9	**88**				88
Family—texture		coarse.loamy	fine	fine.loamy	fine.silty	loamy	none	
	coarse-loamy	**0**	20	13	60	7		
	fine		**67**	5	24	2	2	
	fine-loamy		6	**19**	64	11		
	fine-silty		26	13	**58**			
	loamy		52	11	33	**0**	4	
	none		100				**0**	44

*no information available for this class

The ME composition (Table 3) was markedly inferior in discriminating the difference classes compared to the WE composition. Similar to the WE composition, the ME composition well-discriminated the Suborder class, but confused all of the Great Groups with the Hapludalfs designation. Similarly, the ME composition confused all of the frigid and most of the mesic samples with the thermic temperature regime. Both the WE and ME compositions were largely confused by the semi-active clay activity designation, but the WE composition did a much better job at discriminating the active and super-active classes. The CEC composition showed a similar ability at discriminating the different samples to the ME composition (Table 3). In this sense, the subcompositional coherence of the ME composition (using the CEC subcomposition) was apparent.

**Table 3:** Misclassification matrix from linear discriminant analysis testing the accuracy of 2-PCA model used to describe the Mehlich-III extracted (ME) composition and a 3-PCA model for the CEC subcomposition to correctly assign class memberships of the Alfisols samples among the different Great Group taxonomic designations. Percent correct classifications are shown on the diagonal (in bold) while percent misclassification samples are shown off-diagonal. Accuracy of the CEC subcomposition is given in parenthesis.

Designation	Class	Predicted class						Total accuracy
Suborder		Aqualfs	Udalfs	Ustalfs				
	Aqualfs	**0(0)**	100(100)					
	Udalfs		**99(98)**	1(2)				
	Ustalfs		100(100)	**0(0)**				86(85)
Great Group		Endoaqualfs	Fragiudalfs	Glossudalfs	Hapludalfs	Paleudalfs	Paleustalfs	
	Endoaqualfs	**0(20)**			100(80)			
	Fragiudalfs		**0(0)**		100(88)	0(12)		
	Glossudalfs			**0(0)**	100(100)			
	Hapludalfs	0(1)			**100(90)**	0(4)	0(2)	
	Paleudalfs	0(6)			94(90)	**0(4)**	0(4)	
	Paleustalfs				100(92)		**0(8)**	49(46)
Horizon		Subsurface	Surface					
	Subsurface	**100(96)**	0(4)					
	Surface	100(97)	**0(3)**					62(60)
Family—mineralogy		kaolinitic	mixed	siliceous	smectitic			
	kaolinitic	**0(0)**	100(100)					
	mixed	0(3)	**99(96)**		1(1)			
	siliceous		100(100)	**0(0)**				
	smectitic		100(100)		**0(0)**			67(65)
Family—clay activity		active	none	semiactive	superactive			
	active	**71(80)**	10(8)		18(12)			
	none	62(54)	**6(4)**	0(8)	32(33)			
	semiactive	64(48)	12(2)	**0(7)**	24(43)			
	superactive	64(56)	1(4)	0(8)	**35(32)**			36(39)
Family—temperature		frigid	mesic	thermic				
	frigid	**0(0)**	0(22)	100(78)				
	mesic		**13(16)**	87(84)				
	thermic		4(4)	**96(96)**				52(53)
Family—texture		coarse.loamy	fine	fine.loamy	fine.silty	loamy	none	
	coarse-loamy	**0(40)**	60(20)		40(40)			
	fine		**48(45)**	3(3)	49(49)			
	fine-loamy		97(64)	**0(3)**	3(31)			
	fine-silty	0(3)	36(40)		**64(56)**			
	loamy		22(22)		78(78)	**0(0)**		
	none		72(33)		28(67)		**0(0)**	38(37)

We expected the PSD composition (Table 4) to be the least discriminating composition, given the limited chemical information inherent in this composition. Yet, to our surprise, the PSD composition exhibited a discriminating ability that was, overall, on par with the ME and CEC compositions. Where we expected the PSD composition to excel, in the texture designation (Family name), it performed no better than the other compositions. In general, the different compositions were similarly confused by coarse-loamy, loamy, and fine-loamy textures, but was more accurate in distinguishing the fine-loamy and fine-silty textures.

**Table 4:** Misclassification matrix from linear discriminant analysis testing the accuracy of the 2-PC model used to describe the particle size composition to correctly assign class memberships of the Alfisols samples among the different Great Group taxonomic designations. Percent correct classifications are shown on the diagonal (in bold) while percent misclassification samples are shown off-diagonal.

Designation	Class	Predicted class						Total accuracy
Suborder		Aqualfs	Udalfs	Ustalfs				
	Aqualfs	**0**	100					
	Udalfs		**97**	4				
	Ustalfs		100	**0**				85
Great Group		Endoaqualfs	Fragiudalfs	Glossudalfs	Hapludalfs	Paleudalfs	Paleustalfs	
	Endoaqualfs	**0**			100			
	Fragiudalfs		**19**		81			
	Glossudalfs			**25**	50		25	
	Hapludalfs		12	2	**78**		7	
	Paleudalfs		7		93	**0**		
	Paleustalfs				100		**0**	42
Horizon		Subsurface	Surface					
	Subsurface	**98**	2					
	Surface	100	**0**					62
Family—mineralogy		kaolinitic	mixed	siliceous	smectitic			
	kaolinitic	**50**	50					
	mixed		**97**	2	2			
	siliceous	7	92	**0**				
	smectitic		82	18	**0**			69
Family—clay activity		Active	None	Semiactive	superactive			
	active	**73**	7		20			
	none	16	**53**		32			
	semiactive	69	15	**0**	15			
	superactive	38	29		**33**			47
Family—temperature		frigid	mesic	thermic				
	frigid	**33**	7	60				
	mesic	3	**72**	25				
	thermic	5	33	**62**				60
Family—texture		coarse.loamy	fine	fine.loamy	fine.silty	loamy	none	
	coarse-loamy	**60**	40					
	fine		**4**	13	52	22	9	
	fine-loamy		60	**30**		10		
	fine-silty		15		**85**			
	loamy		33	67		**0**		
	none		67	17	17		**0**	37

## Discussion

This work agrees with other reports [42, 43] showing that multivariate analogies built to represent different soil “types” can do well to capture the natural complexity of soils as articulated by soil classification systems, even in a relatively well-defined soil Order like Alfisols. In this paper, we studied the application of compositional theory to different soil characterization data of Alfisols within the Eastern and Central U.S., and its implications for discriminating soil classification-analogies.

The results showed that the WE composition provided the best discrimination among the different soil classes, both at general and more specific levels of classification. Surprisingly, all of the compositions similarly resolved the soils at the Suborder class, pointing to their value for discriminating soils at more general classification levels. The WE composition was noticeably better at discriminating the samples than the other compositions only one step down the classification hierarchy, at the Great Group level. However, we observed that all of the compositions were confused by the Hapludalfs designation. This may be attributed in part to the inherent ambiguity of this particular class, given that Hapludalfs serves as a “catch-all” designation for unremarkable Udalfs. If so, this represents a potential opportunity for meaningfully revising this designation relative to the composition of interest. At the temperature regime designation (within the Family name), the WE composition correctly classified samples nearly 90% of the time. This result was especially surprising given that the WE composition was missing any information related to soil carbon, a major characteristic typically used to distinguish soils from different climatic regimes. Additional analysis adding soil carbon, nitrogen, and sulfur contents as non-compositional variables (not shown) to the WE composition seemed to have no effect on the misclassification rate for the thermal regime.

Related to this point was the observation that all of the compositions exhibited similar ability to discriminate between surface and subsurface horizons. Here, horizon position was overwhelmingly confused by the subsurface horizon. This probably reflects that fact that the surface horizons for collected Alfisols are typically thin, making it easier to confuse with the subsurface horizons, particularly when diffuse horizon boundaries were apparent. However, adding carbon, nitrogen, and sulfur contents as non-compositional variables to the WE composition greatly improved the accuracy of the horizon position predictions from 68 to 83% (S4 Fig).

CoDA principles provided a clearer theoretical basis for more detailed interpretations of the biplots than we’ve experienced with classical statistical techniques. CoDA also provided a reasonable basis to compare the value of each compositional data set. For example, the collinear relationship in the pH.KCl-Ca-EC subcomposition was sensible, pointing to well-known soil pH buffering mechanisms such as the dissolution of soil CaCO_3_ and release of exchangeable Ca. Witnessing this behavior in the WE composition was reasonable, but, of course, not anticipated in the ME composition because the Mehlich extractant generally overwhelms the soil pH. This pH buffering behavior was clearly more important for discriminating the different soil classes than via acid-extractable solutes or particle size distributions, and perhaps gets us closer to articulating true aqueous chemical behavior in soils. On the other hand, the CEC subcomposition showed the linear relationship between exchangeable Al and Ca cations occupying the exchange phase. At a lower pH, the exchange phase is relatively more enriched in Al^3+^ cations than Ca^2+^ at higher pH. In total, the WE, ME, and CEC compositions emphasized the importance of pH as a dominant soil characteristic.

There are several implications arising from this research. Our results alluded to the value of different compositions emphasizing different ranges of soil behavior. This point is particularly important given that soil data comes in many “shapes and sizes”, representing an important barrier in our efforts to build the described soil analogies. Researchers may use different combinations of destructive (extractions, digests) and non-destructive (XRF, x-ray diffraction) techniques depending on the rarity of the samples, equipment availability, and logistics of transporting soils. To our knowledge, no standard method or protocol is universally suitable for chemically and physically characterizing all soils. This limitation becomes painfully obvious when seeking consensus on the properties important for various soil-mediated reactions, such as contaminant environmental fate or spectroscopic response. Thus, the way forward may involve studying the value of different compositions as opposed to developing a standard characterization protocol for soils, taking advantage of the soils’ subcompositional coherence when considering gaps in one characterization set to another.

These results suggest that classification-analogies could be developed for other NRCS soil types, expecting that inherent or latent structure will similarly emerge from geochemical characterization data as shown in this work for the Alfisols Order. In particular, statistically discriminant analogical models are expected for the more pedomorphologically distinguished taxonomic Orders, such as Mollisols, Ultisols, and Spodosols, and their accompanying Suborder and Great Group sublevels. These results indicate that classification-analogies may be developed for other soil classification systems, such as the previously mentioned WRB, if the specific soil taxonomy of samples can be identified, and the systems are relatively free of arbitrary or poorly defined criteria. In addition, classification analogies may be useful for reconnaissance-related applications, such as developing calibration sets using local soil types for extrapolating soil properties and behavior to remote or otherwise inaccessible soil types. Overall, this research points to the importance of considering soil taxonomy when sampling, providing a means for more explicitly incorporating soil taxonomic information in research.

It is important to note that this work does not include a critical analysis of the NRCS classification system, nor is it the intention of this work to strictly follow conventions among pedologists using the NRCS system in terms of collecting or presenting specific data relative to the classification level. For example, soil nutrient data is typically associated at the Family level in the NRCS classification system, which provides information relevant to land-use, such as whether the land is under agricultural production, etc., because our interest was more related to developing signature earlier on in the hierarchy. This approach was adopted intentionally with the goal of building up a unique soil archive where soil samples could be repeatedly tested for different complex soil processes, such as contaminant sorption or degradation using the same set of soil analogs, and their corresponding multivariate signatures, as opposed to collecting new soil samples when new inquiries arose in terms of complex processes.

## Conclusions

In this paper, we explored the application of CoDA theory to develop highly discriminating soil classification-analogies. We showed that the potential of three different compositions, and one subcomposition, made up of soil characterization data, to accurately predict both taxonomic and location-specific classes of soils. Overall, the WE composition best discriminated the different soil classes.

## Supporting information

S1 Table List of physical and chemical methods used to characterize the soil horizon samples. (DOCX)

S2 Table Variation array for the *clr-*transformed WE composition. (DOCX)

S3 Table Variation array for the *clr-*transformed ME composition. (DOCX)

S4 Table Variation array for the *clr-*transformed PSD composition. (DOCX)

S5 Table Eigenvalues and % explained variance (EV) with the number of components obtained from the robust PCA analysis for the different compositions. (DOCX)

S6 Table Results of normality tests on robust PCA loadings using the Shapiro-Wilk normality tests. (DOCX)

S7 Table MANOVA results on the robust PCA scores for the water-extracted composition. (DOCX)

S8 Table MANOVA results on the robust PCA scores for the Mehlic-III extracted composition. (DOCX)

S9 Table MANOVA results on the robust PCA scores for the particle-size composition. (DOCX)

S1 Fig Missing data matrix for the water-extracted characterization data. (DOCX)

S2 Fig Missing data matrix for the ME characterization data. (DOCX)

S3 Fig Scree plots from the robust PCA of the different compositions. (A) WE composition. (B) ME composition. (C) PSD composition. (D) CEC composition.
(DOCX)

S4 Fig Results from the PCA of the WE composition combining the solid-phase CNS data as non-compositional data. (A) Loadings plot (B) Scores plot.
(DOCX)

## References

[1] Mayo M, Collier ZA, Hoang V, Chappell MA. Uncertainty in Multi-Media Fate and Transport Models: A Case Study for TNT Life Cycle Assessment. Sci Tot Environ. 2014;494–495:104–12. 10.1016/j.scitotenv.2014.06.061 25037048

[2] Cowan CE, Mackay D, Feijtel TCJ, van de Meent D, Guardo AD, Davies J, et al, editors. The Multi-Media Fate Model: A Vital Tool for Predicting the Fate of Chemicals Socieity of Environmental Toxicology and Chemistry (SETAC); 1995 4 14–16, 1994; November 4–5, 1994; Leuven, Belgium and Denver, Colorado.

[3] Mackay D. Multimedia Enviornmental Models: The Fugacity Approach. 2nd ed: CRC Press; 2001.

[4] Chappell MA, Price CL, Miller LF. Solid-phase considerations for the environmental fate of nitrobenzene and triazine munition constituents in soil. Appl Geochem. 2011;26:S330–S3.

[5] Chappell MA. Solid-phase considerations for the environmental fate of TNT and RDX in soil In: Chappell MA, Price CL, George RD, editors. Environmental Chemistry of Explosives and Propellant Compounds in Soils and Marine Systems: Distributed Source Characterization and Remedial Technologies. 1069: American Chemical Society; 2011 p. 1–25.

[6] Wold S, Sjostrom M. SIMCA: A Method for Analyzing Chemical Data in Terms of Similarity and Analogy. Chemometrics: Theory and Application. ACS Symposium Series. 52: American Chemical Society; 1977. p. 243–82.

[7] IUSS Working Group. World Reference Base for Soil Resources 2014, update 2015: International soil classification system for naming soils and creating legends for soil maps Rome: FAO, 2015 Contract No.: 106.

[8] Nachtergaele FO, Spaargaren O, Deckers JA, Ahrens B. New developments in soil classification. Geoderma. 2000;96(4):345–57. 10.1016/S0016-7061(00)00023-9.

[9] De Bakker H. Purposes of soil classification. Geoderma. 1970;4(3):195–208. 10.1016/0016-7061(70)90003-0.

[10] Norris JM. The application of multivriate analysis to soil studies. III. Soil variation. J Soil Sci. 1972;23(1):62–75. 10.1111/j.1365-2389.1972.tb01642.x

[11] Zumberge JE. Prediction of source rock characteristics based on terpane biomarkers in crude oils: A multivariate statistical approach. Geochimica et Cosmochimica Acta. 1987;51(6):1625–37. 10.1016/0016-7037(87)90343-7.

[12] Ballesta RJ, Bueno PC, Rubi JAM, Giménez RG. Pedo-geochemical baseline content levels and soil quality reference values of trace elements in soils from the Mediterranean (Castilla La Mancha, Spain). Cent Eur J Geosci. 2010;2(4):441–54. 10.2478/v10085-010-0028-1

[13] Chappell MA, Seiter JM, Bednar AJ, Price CL, Averett D, Lafferty B, et al Stability of solid-phase selenium species in dredged fly ash after prolonged submersion in a natural river system. Chemosphere. 2013;95:174–81. 10.1016/j.chemosphere.2013.08.061 24095615

[14] Sirven J-B, Salle B, Mauchien P, Lacour J-L, Maurice S, Manhes G. Feasibility study of rock identification at the surface of Mars by remote laser-induced breakdown spectroscopy and three chemometric methods. Journal of Analytical Atomic Spectrometry. 2007;22(12):1471–80. 10.1039/B704868H

[15] Fu J-M, Winchester JW. Inference of nitrogen cycling in three watersheds of northern Florida, USA, by multivariate statistical analysis. Geochimica et Cosmochimica Acta. 1994;58(6):1591–600. 10.1016/0016-7037(94)90561-4.

[16] Ansari AA, Singh IB, Tobschall HJ. Importance of geomorphology and sedimentation processes for metal dispersion in sediments and soils of the Ganga Plain: identification of geochemical domains. Chem Geol. 2000;162(3–4):245–66. 10.1016/S0009-2541(99)00073-X.

[17] Chappell MA, Price CL, Porter BE, Pettway BA, George RD. Differential Kinetics and Temperature Dependence of Abiotic and Biotic Processes Controlling the Environmental Fate of TNT in Simulated Marine Systems. Marine Pollut Bull. 2011;62:1736–43. 10.1016/j.marpolbul.2011.05.026 21683419

[18] Perkey DW, Chappell MA, Seiter JM, Wadman HM. Identification of Sediment Sources to Calumet Harbor and River through Geochemical Techniques USACE-ERDC-CHL, 2017.

[19] Fierer N, Schimel JP, Holden PA. Variations in microbial community composition through two soil depth profiles. Soil Biology and Biochemistry. 2003;35(1):167–76. 10.1016/S0038-0717(02)00251-1.

[20] Singh BK, Munro S, Potts JM, Millard P. Influence of grass species and soil type on rhizosphere microbial community structure in grassland soils. Applied Soil Ecology. 2007;36(2–3):147–55. 10.1016/j.apsoil.2007.01.004.

[21] Cong J, Yang Y, Liu X, Lu H, Liu X, Zhou J, et al Analyses of soil microbial community compositions and functional genes reveal potential consequences of natural forest succession. Scientific Reports. 2015;5:10007 10.1038/srep10007 http://www.nature.com/articles/srep10007#supplementary-information. 25943705 PMC4421864

[22] Ramírez-Guinart O, Vidal M, Rigol A. Univariate and multivariate analysis to elucidate the soil properties governing americium sorption in soils. Geoderma. 2016;269:19–26. 10.1016/j.geoderma.2016.01.026.

[23] Katseanes CK, Chappell MA, Hopkins BG. Multivariate functions for predicting the sorption of 2,4,6-trinitrotoluene (TNT) and 1,3,5-trinitro-1,3,5-tricyclohexane (RDX) among taxonomically distinct soils. J Environ Manag. 2016;182:101–10. 10.1016/j.jenvman.2016.07.043 27454101

[24] Katseanes CK, Chappell MA, Hopkins BG, Durham BS, Price CL, Porter BE, et al Multivariate functions for predicting the degradation kinetics of 2,4,6-trinitrotoluene (TNT) and 1,3,5-trinitro-1,3,5-tricyclohexane (RDX) among taxonomically distinct soils. J Environ Manag. 2017;203:383–90. 10.1016/j.jenvman.2017.08.005 28818710

[25] Katseanes CK. Soil fertility status and degradation of 2,4,6-trinitrotoluene in contaminated soils. Provo, UT: Brigham Young University; 2014.

[26] Pawlowsky-Glahn V, Buccianti A, editors. Compositional Data Analysis: Theory and Applications: John Wiley & Sons, Ltd.; 2011.

[27] Kahle D, Wicham H. ggmap: Spatial Visualization with ggplot2. The R Journal. 2013;5:144–61.

[28] R Development Core Team. R: A language and environment for statistical computing. Vienna, Austria: R Foundation for Statistical Computing; 2018.

[29] Team R. RStudio: Integrated Development for R. Boston, MA: RStudio, Inc.; 2018.

[30] Beaudette DE, Skovlin J, Roecker SM. soilDB: Soil Database Interface. R package version 2.0–1 ed2018. p. A collection of functions for reading data from the USDA-NCSS soil databases.

[31] Reimann C, Filzmoser P, Garrett RG, Dutter R. Principal component analysis (PCA) and factor analysis (FA) Statistical Data Analysis Explained. West Sussex, England: John Wiley & Sons, Ltd.; 2008.

[32] Evangelou VP. Environmental Soil and Water Chemistry: Principles and Applications. New York: John Wiley & Sons, Inc.; 1998.

[33] Palarea-Albaladejo J, Martin-Fernandez JA. zCompositions—R package for multivariate imputation of left-censored data under a compositional approach. Chemom Intell Lab Syst. 2015;143:85–96.

[34] van den Boogaart G, Tolosana-Delgato R, Bren M. compositions: Compositional Data Analysis. R package version 1.40–2 ed2018.

[35] Templ M, Hron K, Filzmoser P. robCompositions: an R-package for robust statistical analysis of compositional data Chichester, UK: John Wiley & Sons, Inc.; 2011.

[36] Jackson DA. Stopping Rules in Principal Components Analysis: A Comparison of Heuristical and Statistical Approaches. Ecology. 1993;74(8):2204–14. 10.2307/1939574

[37] Venables WN, Ripley BD. Modern Applied Statistics with S. 4th ed. New York: Springer; 2002.

[38] John A. Logratios and Natural Laws in Compositional Data Analysis. Mathematical Geology. 1999;31:563–80.

[39] van den Boogaart G, Tolosana-Delgato R, Bren M. Analyzing Compositional Data with R. New York: Springer; 2013.

[40] Aitchison J, Greenacre M. Biplots of compositional data. Journal of the Royal Statistical Society: Series C (Applied Statistics). 2002;51(4):375–92. 10.1111/1467-9876.00275

[41] Hernández Suárez M, Molina Pérez D, Rodríguez-Rodríguez EM, Díaz Romero C, Espinosa Borreguero F, Galindo-Villardón P. The Compositional HJ-Biplot—A New Approach to Identifying the Links among Bioactive Compounds of Tomatoes. International Journal of Molecular Sciences. 2016;17(11):1828 10.3390/ijms17111828 PMC5133829. 27827839 PMC5133829

[42] Hughes P, McBratney AB, Huang J, Minasny B, Micheli E, Hempel J. Comparisons between USDA Soil Taxonomy and the Australian Soil Classification System I: Data harmonization, calculation of taxonomic distance and inter-taxa variation. Geoderma. 2017;307:198–209. 10.1016/j.geoderma.2017.08.009.

[43] Hughes P, McBratney A, Huang J, Minasny B, Hempel J, Palmer DJ, et al Creating a novel comprehensive soil classification system by sequentially adding taxa from existing systems. Geoderma Regional. 2017;11:123–40. 10.1016/j.geodrs.2017.10.004.

