# Supplementary material for: Building geochemically based quantitative analogies from soil classification systems using different compositional datasets
Source: PLoS One. 2019 Feb 19;14(2):e0212214. doi: 10.1371/journal.pone.0212214 (PMC6380586; doi:10.1371/journal.pone.0212214)
Supplement: S1 Table — (DOCX) [file pone.0212214.s001.docx]

| **Measurement** | **Method** | **Reference** |
| --- | --- | --- |
| pH/EC | Separate extractions in water, 1 *M* KCl, and 20 *mM* CaCl_2_. Extractions were conducted using 5 g soil and 5 mL solution in propylene test tubes, shaken at low speed on a reciprocating shaker for 4 h, and settled on bench at room temperature for 1 h. pH and EC were measured on the overlying solution using Fisherbrand combination pH and conductivity probes, respectively. Probes were calibrated as recommended by manufacturer. | (Adapted from Rhoades, 1996; Adapted from Thomas, 1996) |
| particle size distribution | Modified pipet method that includes measurement of particulate organic matter (POM). Soils were suspended in 0.5% NaPO_3_ (sodium hexametaphosphate) solution in 250 mL Nalgene bottles and shaken for approx. 16 h using a reciprocating shaker. Suspension was sieved through stacked 0.5 mm (no. 35) and 0.053 mm (no. 270) mesh sieves. Suspension that passed through the 0.053 mm sieve were collected in a plastic bucket. This suspension was transferred to a 1 L beaker and silt and clay via pipette method during settling. Particles collected on the 0.053 mm sieve (representing both the sand and POM fractions) were dried, weighed, heated at 450 deg C for 4 h, and then reweighted. %sand was calculated by difference after heating. | (Kettler and Doran, 2001) |
| Elemental analysis | Water extraction (2.5 g soil in 25 mL water), shaken at low speed on a reciprocating shaker for 4 h, and centrifuged for 10 min at 9681 *xg*. Supernatant analyzed via ICP-OES-MS following EPA Method 6010. | (USEPA, 2014) |
| Soil extractable NH_4_-N | Suspended 2.5 g soil in 25 mL 1 *M* KCl and shook at low speed on a reciprocating shaker for 4 h. Centrifuged for 10 min at 9681 *xg*. Collected supernatant and NH_4_-N analyzed using Berthot colorimetric assay. | (Keeney and Nelson, 1982) |
| Solution NO_2_-N, NO_3_-N, and common anions, including Cl, PO_4_, and SO_4_. | Water extraction (2.5 g soil in 25 mL water), shaking for 4 hr, and centrifuge for 10 min at 9681 *xg*. NO_2_-N analyzed using Griess-Hosvay assay; NO_3_-N analyzed using Griess-Hosvay assay with Cd reduction. Other anions analyzed using ion chromatography. | (Bremner, 1965; Mulvaney, 1996) |
| Soil solid-phase organic and inorganic carbon; soil total nitrogen; soil total sulfur. | Catalytic combustion using a LECO (Saint Joseph, MI, USA) CHN628S series instrument. | ASTM (2016, 2017) |
| Extractable metals and cations | Mehlich III extraction using 2.5 g soil in 20 mL extractant, shaking for 4 hr, and centrifuge for 10 min at 9681 *xg*. Supernatant analyzed via ICP-OES-MS following EPA Method 6010. | (Amacher, 1996) |
|  |  |  |
