# Supplementary material for: Building geochemically based quantitative analogies from soil classification systems using different compositional datasets
Source: PLoS One. 2019 Feb 19;14(2):e0212214. doi: 10.1371/journal.pone.0212214 (PMC6380586; doi:10.1371/journal.pone.0212214)
Supplement: S2 Table — (DOCX) [file pone.0212214.s002.docx]

|  | NH4.N | Cl | NO3 | SO4 | Ba.w | Ca.w | Co.w | Cu.w | Fe.w | Pb.w | Mg.w | Mn.w | Ni.w | K.w | Na.w | V.w | Zn.w | pH.H2O | pH.KCl | pH.CaCl2 | EC |
| --- | --- | --- | --- | --- | --- | --- | --- | --- | --- | --- | --- | --- | --- | --- | --- | --- | --- | --- | --- | --- | --- |
| NH4.N |  | 10.547 | 9.054 | 7.147 | 4.568 | 2.672 | 3.913 | 3.139 | 3.140 | 4.045 | 3.507 | 1.794 | 2.897 | 4.661 | 5.727 | 3.466 | 7.509 | 7.672 | 7.749 | 10.152 | 3.214 |
| Cl | 10.547 |  | 3.309 | 1.151 | 0.856 | 4.564 | 1.235 | 2.624 | 1.543 | 1.795 | 3.518 | 3.600 | 2.017 | 1.447 | 0.235 | 1.361 | 0.343 | 2.622 | 4.417 | 3.928 | 2.613 |
| NO3 | 9.054 | 3.309 |  | 3.622 | 2.610 | 3.091 | 3.236 | 2.315 | 4.096 | 1.958 | 5.157 | 3.949 | 3.658 | 3.133 | 2.684 | 2.237 | 2.072 | 9.172 | 13.435 | 10.446 | 3.343 |
| SO4 | 7.147 | 1.151 | 3.622 |  | 0.837 | 1.927 | 0.902 | 1.505 | 1.132 | 0.589 | 2.465 | 1.799 | 0.891 | 0.842 | 0.672 | 0.960 | 0.632 | 3.577 | 6.328 | 3.662 | 1.145 |
| Ba.w | 4.568 | 0.856 | 2.610 | 0.837 |  | 1.045 | 0.710 | 0.605 | 0.396 | 0.559 | 0.946 | 0.942 | 0.614 | 0.475 | 0.294 | 0.629 | 0.498 | 5.363 | 7.493 | 5.969 | 0.991 |
| Ca.w | 2.672 | 4.564 | 3.091 | 1.927 | 1.045 |  | 1.929 | 1.428 | 1.438 | 1.030 | 0.452 | 0.768 | 1.458 | 1.047 | 1.755 | 1.270 | 1.691 | 10.617 | 15.012 | 12.674 | 0.519 |
| Co.w | 3.913 | 1.235 | 3.236 | 0.902 | 0.710 | 1.929 |  | 0.749 | 0.742 | 0.932 | 1.034 | 1.095 | 0.500 | 1.022 | 0.638 | 0.783 | 0.634 | 5.002 | 6.592 | 4.787 | 1.226 |
| Cu.w | 3.139 | 2.624 | 2.315 | 1.505 | 0.605 | 1.428 | 0.749 |  | 0.795 | 0.526 | 1.109 | 1.045 | 0.385 | 0.579 | 1.129 | 0.980 | 0.653 | 7.899 | 9.634 | 7.306 | 0.865 |
| Fe.w | 3.140 | 1.543 | 4.096 | 1.132 | 0.396 | 1.438 | 0.742 | 0.795 |  | 0.842 | 1.285 | 0.833 | 0.639 | 0.558 | 0.521 | 0.496 | 0.812 | 5.713 | 7.295 | 7.008 | 0.754 |
| Pb.w | 4.045 | 1.795 | 1.958 | 0.589 | 0.559 | 1.030 | 0.932 | 0.526 | 0.842 |  | 1.456 | 0.542 | 1.000 | 0.499 | 0.726 | 0.639 | 0.394 | 6.266 | 8.398 | 7.404 | 0.548 |
| Mg.w | 3.507 | 3.518 | 5.157 | 2.465 | 0.946 | 0.452 | 1.034 | 1.109 | 1.285 | 1.456 |  | 0.914 | 0.638 | 0.738 | 1.383 | 1.033 | 2.363 | 7.178 | 9.545 | 7.950 | 0.786 |
| Mn.w | 1.794 | 3.600 | 3.949 | 1.799 | 0.942 | 0.768 | 1.095 | 1.045 | 0.833 | 0.542 | 0.914 |  | 0.938 | 0.917 | 1.218 | 1.116 | 1.657 | 7.089 | 10.791 | 8.434 | 0.705 |
| Ni.w | 2.897 | 2.017 | 3.658 | 0.891 | 0.614 | 1.458 | 0.500 | 0.385 | 0.639 | 1.000 | 0.638 | 0.938 |  | 1.080 | 1.159 | 0.652 | 1.257 | 5.633 | 7.254 | 4.383 | 1.070 |
| K.w | 4.661 | 1.447 | 3.133 | 0.842 | 0.475 | 1.047 | 1.022 | 0.579 | 0.558 | 0.499 | 0.738 | 0.917 | 1.080 |  | 0.559 | 0.763 | 0.563 | 6.794 | 9.668 | 8.345 | 0.472 |
| Na.w | 5.727 | 0.235 | 2.684 | 0.672 | 0.294 | 1.755 | 0.638 | 1.129 | 0.521 | 0.726 | 1.383 | 1.218 | 1.159 | 0.559 |  | 0.642 | 0.257 | 5.189 | 7.759 | 5.161 | 0.688 |
| V.w | 3.466 | 1.361 | 2.237 | 0.960 | 0.629 | 1.270 | 0.783 | 0.980 | 0.496 | 0.639 | 1.033 | 1.116 | 0.652 | 0.763 | 0.642 |  | 0.747 | 5.390 | 6.190 | 5.564 | 1.098 |
| Zn.w | 7.509 | 0.343 | 2.072 | 0.632 | 0.498 | 1.691 | 0.634 | 0.653 | 0.812 | 0.394 | 2.363 | 1.657 | 1.257 | 0.563 | 0.257 | 0.747 |  | 5.036 | 6.470 | 5.481 | 1.141 |
| pH.H2O | 7.672 | 2.622 | 9.172 | 3.577 | 5.363 | 10.617 | 5.002 | 7.899 | 5.713 | 6.266 | 7.178 | 7.089 | 5.633 | 6.794 | 5.189 | 5.390 | 5.036 |  | 0.514 | 0.443 | 9.117 |
| pH.KCl | 7.749 | 4.417 | 13.435 | 6.328 | 7.493 | 15.012 | 6.592 | 9.634 | 7.295 | 8.398 | 9.545 | 10.791 | 7.254 | 9.668 | 7.759 | 6.190 | 6.470 | 0.514 |  | 0.700 | 11.430 |
| pH.CaCl2 | 10.152 | 3.928 | 10.446 | 3.662 | 5.969 | 12.674 | 4.787 | 7.306 | 7.008 | 7.404 | 7.950 | 8.434 | 4.383 | 8.345 | 5.161 | 5.564 | 5.481 | 0.443 | 0.700 |  | 8.928 |
| EC | 3.214 | 2.613 | 3.343 | 1.145 | 0.991 | 0.519 | 1.226 | 0.865 | 0.754 | 0.548 | 0.786 | 0.705 | 1.070 | 0.472 | 0.688 | 1.098 | 1.141 | 9.117 | 11.430 | 8.928 |  |
