# Supplementary material for: Building geochemically based quantitative analogies from soil classification systems using different compositional datasets
Source: PLoS One. 2019 Feb 19;14(2):e0212214. doi: 10.1371/journal.pone.0212214 (PMC6380586; doi:10.1371/journal.pone.0212214)
Supplement: S3 Table — (DOCX) [file pone.0212214.s003.docx]

|  | Al.m | Ba.m | Ca.m | Co.m | Cu.m | Fe.m | Pb.m | Mg.m | Mn.m | Ni.m | K.m | Na.m | V.m | Zn.m | CEC |
| --- | --- | --- | --- | --- | --- | --- | --- | --- | --- | --- | --- | --- | --- | --- | --- |
| Al.m |  | 0.481 | 1.968 | 0.705 | 0.940 | 0.439 | 0.641 | 1.296 | 1.106 | 0.723 | 0.794 | 0.234 | 0.812 | 0.794 | 1.213 |
| Ba.m | 0.481 |  | 1.037 | 0.729 | 0.610 | 0.369 | 0.533 | 0.946 | 1.019 | 0.638 | 0.493 | 0.302 | 0.629 | 0.487 | 0.724 |
| Ca.m | 1.968 | 1.037 |  | 1.727 | 1.470 | 1.446 | 1.030 | 0.446 | 0.768 | 1.496 | 1.086 | 1.860 | 1.521 | 1.845 | 0.053 |
| Co.m | 0.705 | 0.729 | 1.727 |  | 0.795 | 0.793 | 0.932 | 1.064 | 0.948 | 0.805 | 1.110 | 0.696 | 0.849 | 0.983 | 1.188 |
| Cu.m | 0.940 | 0.610 | 1.470 | 0.795 |  | 0.794 | 0.526 | 1.165 | 1.145 | 0.591 | 0.565 | 1.154 | 0.800 | 0.785 | 1.280 |
| Fe.m | 0.439 | 0.369 | 1.446 | 0.793 | 0.794 |  | 0.814 | 1.162 | 0.901 | 0.653 | 0.562 | 0.526 | 0.501 | 0.781 | 1.131 |
| Pb.m | 0.641 | 0.533 | 1.030 | 0.932 | 0.526 | 0.814 |  | 1.438 | 0.542 | 1.100 | 0.499 | 0.666 | 0.803 | 0.394 | 0.832 |
| Mg.m | 1.296 | 0.946 | 0.446 | 1.064 | 1.165 | 1.162 | 1.438 |  | 0.960 | 0.870 | 0.738 | 1.344 | 1.136 | 2.432 | 0.280 |
| Mn.m | 1.106 | 1.019 | 0.768 | 0.948 | 1.145 | 0.901 | 0.542 | 0.960 |  | 1.003 | 1.035 | 1.337 | 1.358 | 1.657 | 0.767 |
| Ni.m | 0.723 | 0.638 | 1.496 | 0.805 | 0.591 | 0.653 | 1.100 | 0.870 | 1.003 |  | 1.061 | 1.215 | 0.548 | 1.371 | 0.901 |
| K.m | 0.794 | 0.493 | 1.086 | 1.110 | 0.565 | 0.562 | 0.499 | 0.738 | 1.035 | 1.061 |  | 0.586 | 0.747 | 0.563 | 0.472 |
| Na.m | 0.234 | 0.302 | 1.860 | 0.696 | 1.154 | 0.526 | 0.666 | 1.344 | 1.337 | 1.215 | 0.586 |  | 0.683 | 0.254 | 1.191 |
| V.m | 0.812 | 0.629 | 1.521 | 0.849 | 0.800 | 0.501 | 0.803 | 1.136 | 1.358 | 0.548 | 0.747 | 0.683 |  | 0.688 | 0.963 |
| Zn.m | 0.794 | 0.487 | 1.845 | 0.983 | 0.785 | 0.781 | 0.394 | 2.432 | 1.657 | 1.371 | 0.563 | 0.254 | 0.688 |  | 1.168 |
| CEC | 1.213 | 0.724 | 0.053 | 1.188 | 1.280 | 1.131 | 0.832 | 0.280 | 0.767 | 0.901 | 0.472 | 1.191 | 0.963 | 1.168 |  |
