# Supplementary material for: Building geochemically based quantitative analogies from soil classification systems using different compositional datasets
Source: PLoS One. 2019 Feb 19;14(2):e0212214. doi: 10.1371/journal.pone.0212214 (PMC6380586; doi:10.1371/journal.pone.0212214)
Supplement: S4 Table — (DOCX) [file pone.0212214.s004.docx]

| Variables | Sand | Silt | Clay |
| --- | --- | --- | --- |
| Sand | 0 | 3.892705 | 3.230026 |
| Silt | 3.892705 | 0 | 0.130268 |
| Clay | 3.230026 | 0.130268 | 0 |
