# Supplementary material for: Building geochemically based quantitative analogies from soil classification systems using different compositional datasets
Source: PLoS One. 2019 Feb 19;14(2):e0212214. doi: 10.1371/journal.pone.0212214 (PMC6380586; doi:10.1371/journal.pone.0212214)
Supplement: S5 Table — (DOCX) [file pone.0212214.s005.docx]

| **Composition** | **WE** | | **ME** | | **PSD** | | **CEC** | |
| --- | --- | --- | --- | --- | --- | --- | --- | --- |
| Components | Eigenvalue | %EV | Eigenvalue | %EV | Eigenvalue | %EV | Eigenvalue | %EV |
| 1 | 21.39535 | 64.3 | 1.765136 | 30.1 | 2.042679 | 97.2 | 1.248294 | 67.5 |
| 2 | 4.947586 | 14.9 | 1.191585 | 20.3 | 0.059032 | 2.8 | 0.296942 | 16.1 |
| 3 | 1.764926 | 5.3 | 0.792081 | 13.5 |  |  | 0.171384 | 9.3 |
| 4 | 1.30217 | 3.9 | 0.62729 | 10.7 |  |  | 0.132056 | 7.1 |
| 5 | 1.032934 | 3.1 | 0.358629 | 6.1 |  |  |  |  |
| 6 | 0.70775 | 2.1 | 0.310055 | 5.3 |  |  |  |  |
| 7 | 0.438243 | 1.3 | 0.238675 | 4.1 |  |  |  |  |
| 8 | 0.362681 | 1.1 | 0.187877 | 3.2 |  |  |  |  |
| 9 | 0.276629 | 0.8 | 0.133769 | 2.3 |  |  |  |  |
| 10 | 0.253463 | 0.8 | 0.103258 | 1.8 |  |  |  |  |
| 11 | 0.188831 | 0.6 | 0.067031 | 1.1 |  |  |  |  |
| 12 | 0.12692 | 0.4 | 0.048769 | 0.8 |  |  |  |  |
| 13 | 0.099753 | 0.3 | 0.038752 | 0.7 |  |  |  |  |
| 14 | 0.088978 | 0.3 | 0.001114 | 0 |  |  |  |  |
| 15 | 0.080132 | 0.2 | 1.765136 | 30.1 |  |  |  |  |
| 16 | 0.065444 | 0.2 | 1.191585 | 20.3 |  |  |  |  |
| 17 | 0.053829 | 0.2 | 0.792081 | 13.5 |  |  |  |  |
| 18 | 0.042662 | 0.1 | 0.62729 | 10.7 |  |  |  |  |
| 19 | 0.02357 | 0.1 | 0.358629 | 6.1 |  |  |  |  |
| 20 | 0.013589 | 0 |  |  |  |  |  |  |
| 21 | 0.019519 | 0.0 |  |  |  |  |  |  |
