# Supplementary material for: Building geochemically based quantitative analogies from soil classification systems using different compositional datasets
Source: PLoS One. 2019 Feb 19;14(2):e0212214. doi: 10.1371/journal.pone.0212214 (PMC6380586; doi:10.1371/journal.pone.0212214)
Supplement: S6 Table — (DOCX) [file pone.0212214.s006.docx]

| Composition | Variable loading | W-statistic | p-value* | H0 vs. Ha |
| --- | --- | --- | --- | --- |
| Water-extract | NH4.N | 0.910008594 | 0.054931673 | H0 |
|  | Cl | 0.943669484 | 0.257296605 | H0 |
|  | NO3 | 0.835376666 | 0.002402798 | H0 |
|  | SO4 | 0.949643838 | 0.335402853 | H0 |
|  | Al.w | 0.986515176 | 0.987409518 | H0 |
|  | Ba.w | 0.959274914 | 0.501639663 | H0 |
|  | Co.w | 0.937281823 | 0.192462039 | H0 |
|  | Cu.w | 0.981784431 | 0.948520707 | H0 |
|  | Fe.w | 0.915052702 | 0.069144652 | H0 |
|  | Pb.w | 0.983410378 | 0.965597728 | H0 |
|  | Mg.w | 0.957532574 | 0.467886504 | H0 |
|  | Mn.w | 0.97168232 | 0.769954869 | H0 |
|  | Ni.w | 0.873186819 | 0.010945883 | H0 |
|  | K.w | 0.940964226 | 0.227672752 | H0 |
|  | Na.w | 0.897487443 | 0.031289405 | H0 |
|  | Ti.w | 0.944134814 | 0.262736191 | H0 |
|  | V.w | 0.974139119 | 0.821781151 | H0 |
|  | Zn.w | 0.971707789 | 0.770506929 | H0 |
|  | pH.KCl | 0.955592481 | 0.432153366 | H0 |
|  | pH.CaCl2 | 0.93828187 | 0.201475102 | H0 |
|  | EC | 0.951794844 | 0.368138868 | H0 |
|  | pH.Ca.var | 0.911084688 | 0.057687405 | H0 |
| Mehilich-III | Al.m | 0.977830031 | 0.960274864 | H0 |
|  | Ba.m | 0.967056477 | 0.83501196 | H0 |
|  | Ca.m | 0.976361924 | 0.948200453 | H0 |
|  | Co.m | 0.865966726 | 0.036845532 | H0 |
|  | Cu.m | 0.957038137 | 0.674160802 | H0 |
|  | Fe.m | 0.826221223 | 0.010729976 | H0 |
|  | Pb.m | 0.957792472 | 0.686624904 | H0 |
|  | Mg.m | 0.927578265 | 0.282098015 | H0 |
|  | Mn.m | 0.902661715 | 0.123280605 | H0 |
|  | Ni.m | 0.893248863 | 0.090046192 | H0 |
|  | K.m | 0.876077579 | 0.051117291 | H0 |
|  | Na.m | 0.96322211 | 0.775595443 | H0 |
|  | V.m | 0.927266609 | 0.279248942 | H0 |
|  | Zn.m | 0.975483394 | 0.940123109 | H0 |
|  | CEC | 0.792275354 | 0.004011838 | H0 |
