# Supplementary material for: Building geochemically based quantitative analogies from soil classification systems using different compositional datasets
Source: PLoS One. 2019 Feb 19;14(2):e0212214. doi: 10.1371/journal.pone.0212214 (PMC6380586; doi:10.1371/journal.pone.0212214)
Supplement: S7 Table — (DOCX) [file pone.0212214.s007.docx]

| Class | Wilks | F | Probability |
| --- | --- | --- | --- |
| Suborder | 0.216310927 | 14.8419016 | 1.25578E-65 |
|  |  |  |  |
| Great Group | 0.02285597 | 14.60152429 | 2.7253E-156 |
|  |  |  |  |
| Surface/subsurface | 0.45509089 | 15.50870503 | 2.46569E-35 |
|  |  |  |  |
| Family-mineralogy | 0.103203252 | 14.59959297 | 1.61764E-95 |
|  |  |  |  |
| Family-clay activity | 0.020744568 | 23.88396255 | 6.4018E-122 |
|  |  |  |  |
| Family-temperature | 0.063031926 | 38.49601771 | 2.7658E-135 |
|  |  |  |  |
| Family-texture |  |  |  |
