# Supplementary material for: Building geochemically based quantitative analogies from soil classification systems using different compositional datasets
Source: PLoS One. 2019 Feb 19;14(2):e0212214. doi: 10.1371/journal.pone.0212214 (PMC6380586; doi:10.1371/journal.pone.0212214)
Supplement: S8 Table — (DOCX) [file pone.0212214.s008.docx]

| Class | Wilks | F | Probability |
| --- | --- | --- | --- |
| Suborder | 0.215490986 | 22.91905761 | 9.11227E-75 |
|  |  |  |  |
| Great Group | 0.101036439 | 11.60359322 | 1.68966E-94 |
|  |  |  |  |
| Surface/subsurface | 0.960798687 | 0.813100793 | 0.654738201 |
|  |  |  |  |
| Family-mineralogy | 0.482521611 | 5.453004809 | 1.21291E-23 |
|  |  |  |  |
| Family-clay activity | 0.337879172 | 6.121815111 | 5.69122E-26 |
|  |  |  |  |
| Family-temperature | 0.524072791 | 7.572550598 | 7.99586E-25 |
|  |  |  |  |
| Family-texture |  |  |  |
