# Supplementary material for: Building geochemically based quantitative analogies from soil classification systems using different compositional datasets
Source: PLoS One. 2019 Feb 19;14(2):e0212214. doi: 10.1371/journal.pone.0212214 (PMC6380586; doi:10.1371/journal.pone.0212214)
Supplement: S9 Table — (DOCX) [file pone.0212214.s009.docx]

|  | Wilks | F | Probability. |  |
| --- | --- | --- | --- | --- |
| Suborder | 0.837198673 | 3.716534343 | 0.006387044 |  |
|  |  |  |  |  |
| Great Group | 0.583081978 | 4.767678599 | 5.90168E-06 |  |
|  |  |  |  |  |
| Surface/subsurface | 0.982509654 | 0.720969002 | 0.489374654 |  |
|  |  |  |  |  |
| Family-minearology | 0.641415062 | 6.54700363 | 3.42241E-06 |  |
|  |  |  |  |  |
| Family-clay activity | 0.735719691 | 3.261776482 | 0.005287942 |  |
|  |  |  |  |  |
| Family-temperature | 0.657308056 | 9.337315338 | 8.29224E-07 |  |
|  |  |  |  |  |
| Family-texture |  |  |  |  |
