# Supplementary figures and images for: Building geochemically based quantitative analogies from soil classification systems using different compositional datasets
Source: PLoS One. 2019 Feb 19;14(2):e0212214. doi: 10.1371/journal.pone.0212214 (PMC6380586; doi:10.1371/journal.pone.0212214)

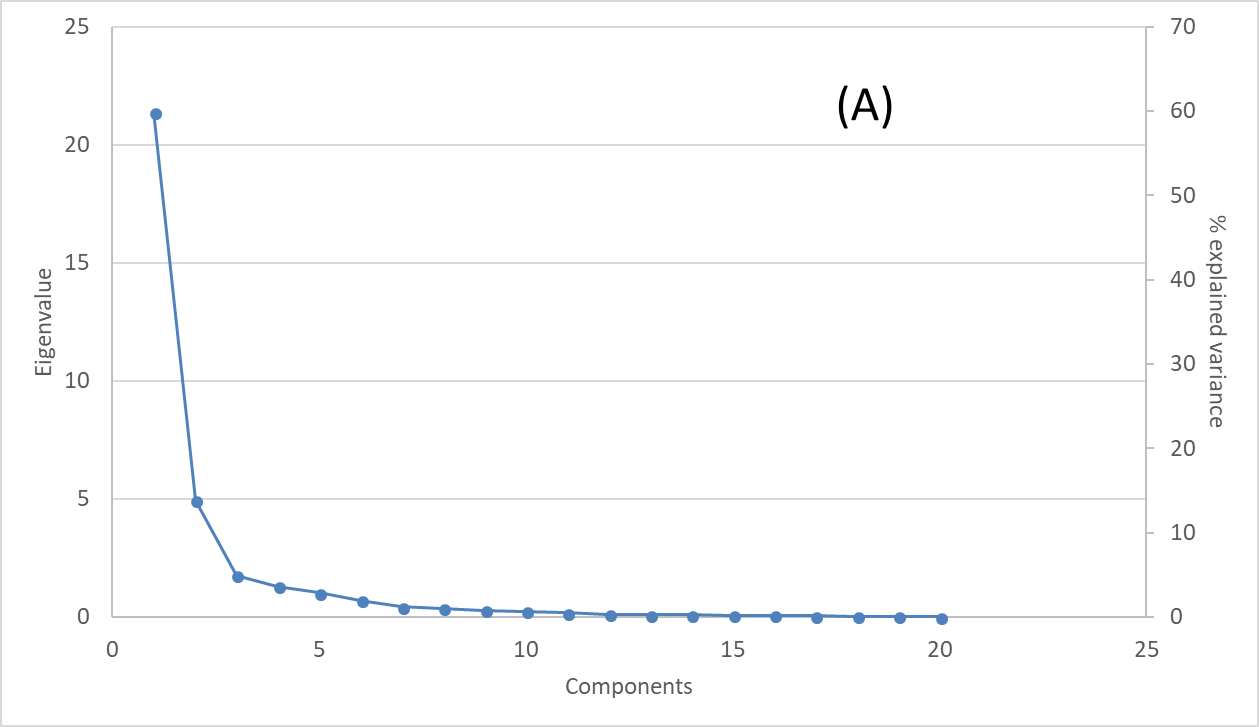

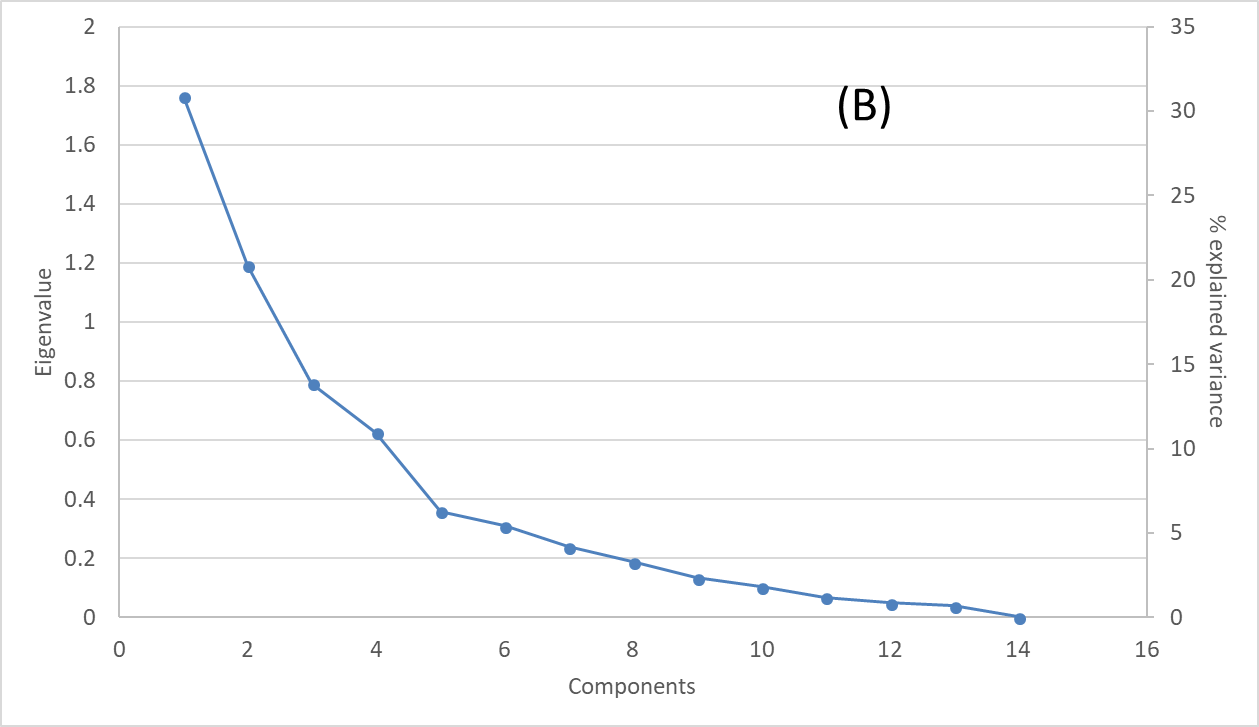

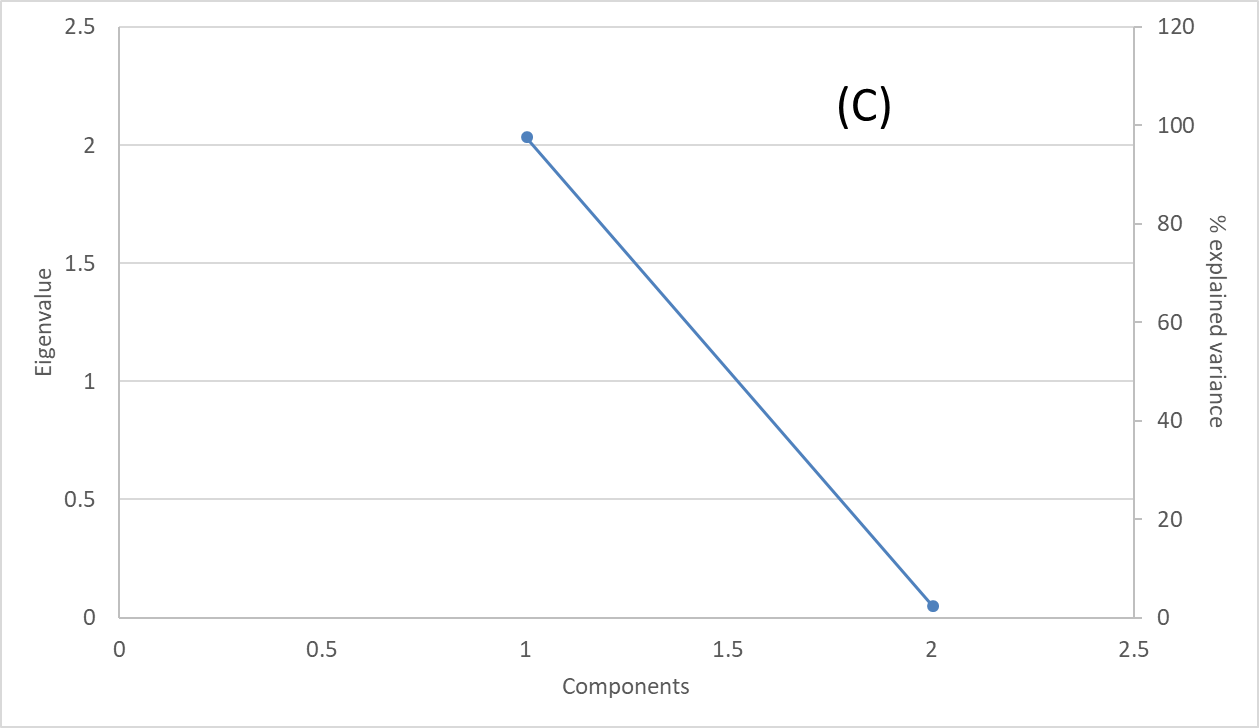

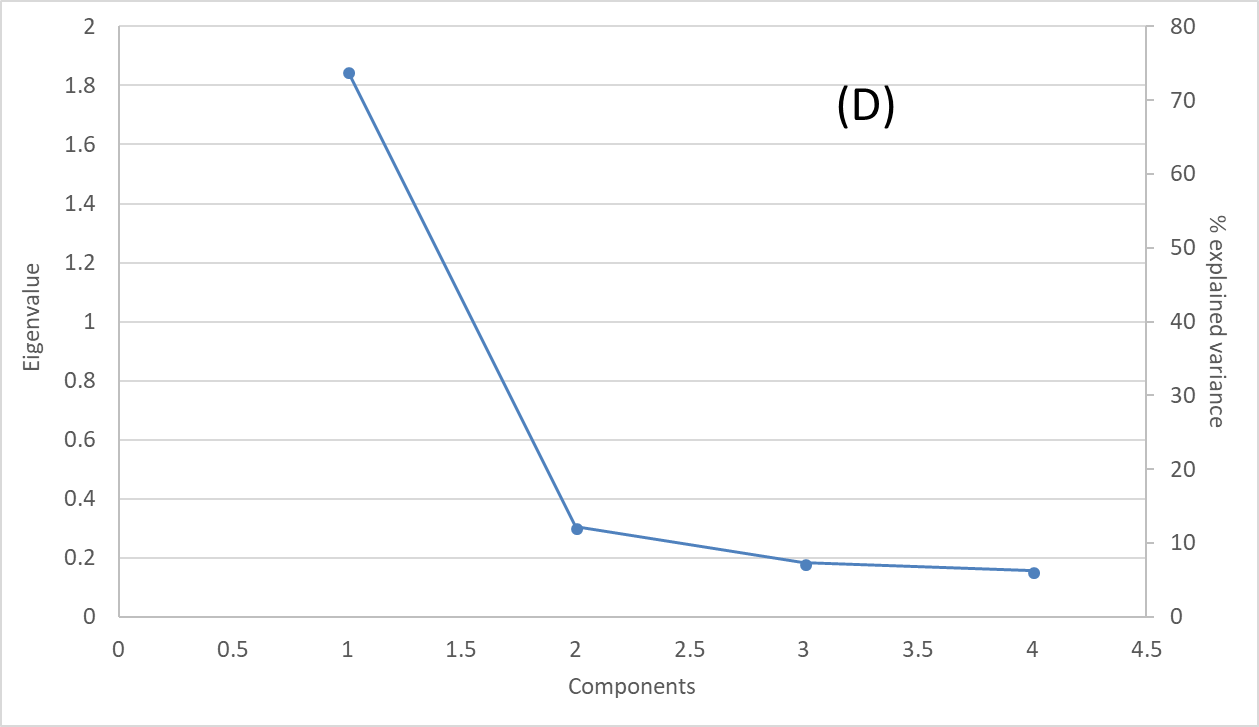

Supplement: S3 Fig — (A) WE composition. (B) ME composition. (C) PSD composition. (D) CEC composition. (DOCX) [file pone.0212214.s012.docx]
