# Supplementary material for: Building geochemically based quantitative analogies from soil classification systems using different compositional datasets
Source: PLoS One. 2019 Feb 19;14(2):e0212214. doi: 10.1371/journal.pone.0212214 (PMC6380586; doi:10.1371/journal.pone.0212214)
Supplement: Supplementary file 11 [file pone.0212214.s010.docx]

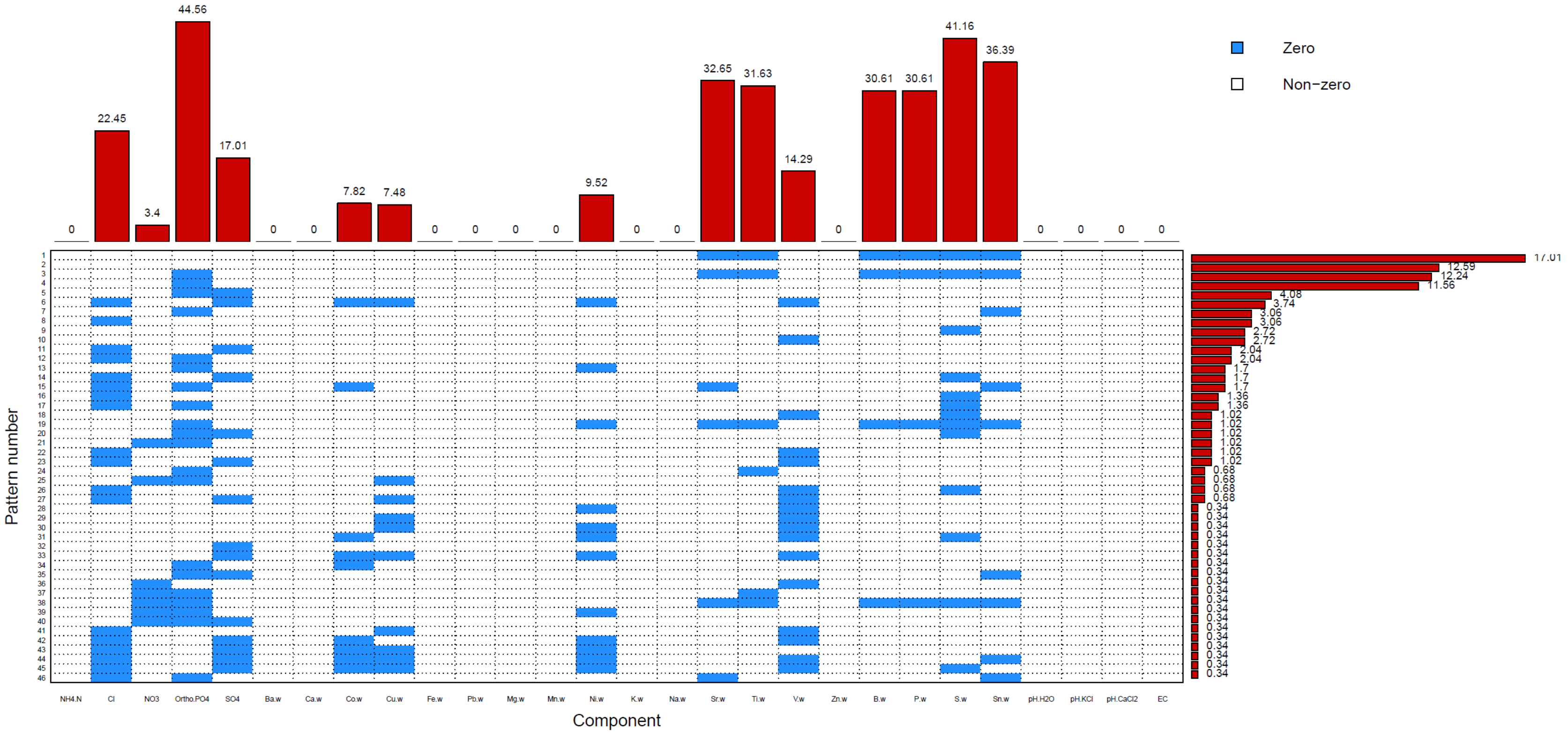

Zero
Non−zero
Pattern number
Component
NH4.N
Cl
NO3
Ortho.PO4
SO4
Ba.w
Ca.w
Co.w
Cu.w
Fe.w
Pb.w
Mg.w
Mn.w
Ni.w
K.w
Na.w
Sr.w
Ti.w
V.w
Zn.w
B.w
P.w
S.w
Sn.w
pH.H2O
pH.KCl
pH.CaCl2
EC
