# Supplementary material for: Building geochemically based quantitative analogies from soil classification systems using different compositional datasets
Source: PLoS One. 2019 Feb 19;14(2):e0212214. doi: 10.1371/journal.pone.0212214 (PMC6380586; doi:10.1371/journal.pone.0212214)
Supplement: Supplementary file 12 [file pone.0212214.s011.docx]

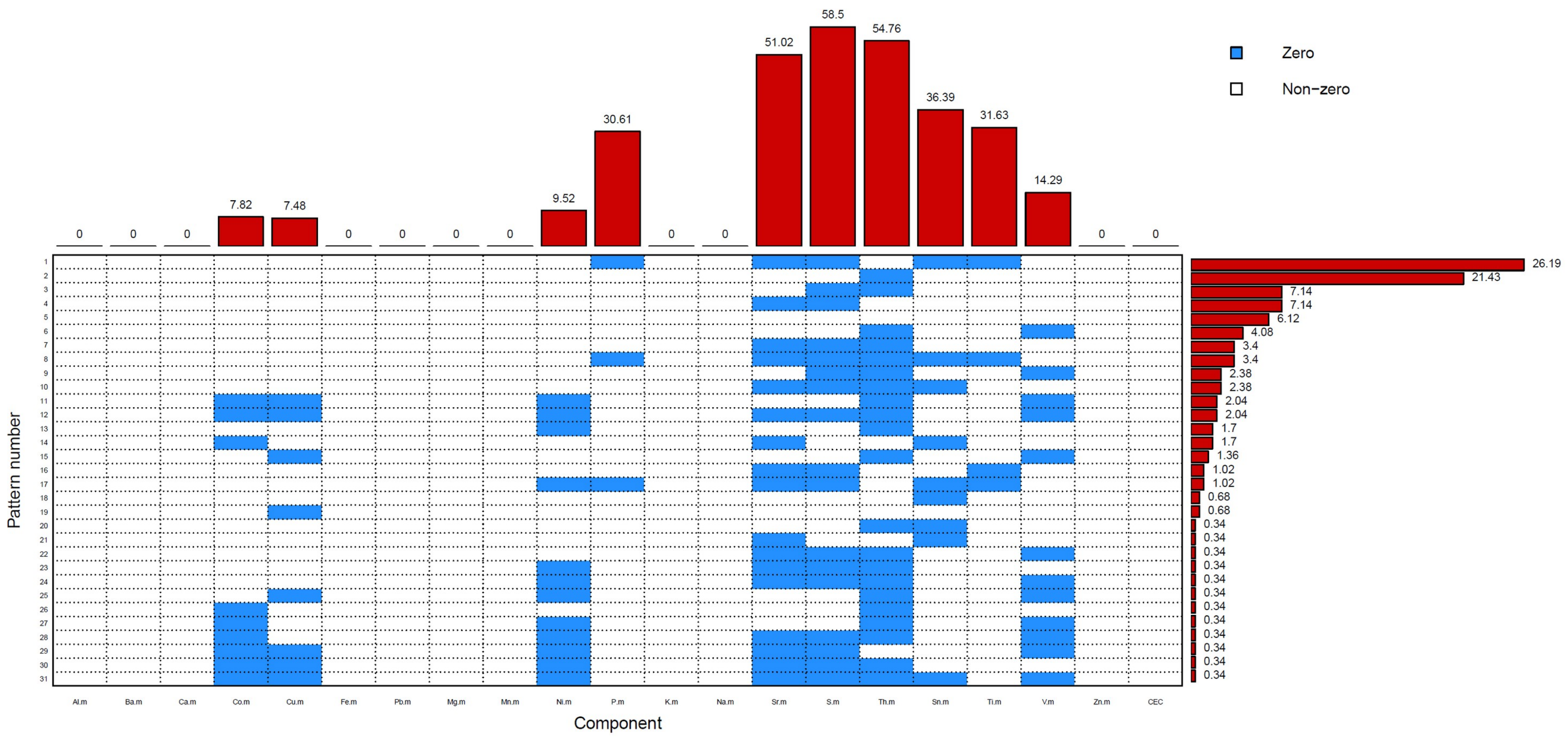
Zero
Non-zero
0
0
0
7.82
7.48
0
0
0
0
9.52
30.61
0
0
51.02
58.5
54.76
36.39
31.63
14.29
0
0
Pattern number
Component
Al.m
Ba.m
Ca.m
Co.m
Cu.m
Fe.m
Pb.m
Mg.m
Mn.m
Ni.m
P.m
K.m
Na.m
Sr.m
S.m
Th.m
Sn.m
Ti.m
V.m
Zn.m
CEC
26.19
21.43
7.14
7.14
6.12
4.08
3.4
3.4
2.38
2.38
2.04
2.04
1.7
1.7
1.36
1.02
1.02
0.68
0.68
0.34
0.34
0.34
0.34
0.34
0.34
0.34
0.34
0.34
0.34
0.34
0.34
0.34
