# Supplementary material for: Building geochemically based quantitative analogies from soil classification systems using different compositional datasets
Source: PLoS One. 2019 Feb 19;14(2):e0212214. doi: 10.1371/journal.pone.0212214 (PMC6380586; doi:10.1371/journal.pone.0212214)
Supplement: Supplementary file 13 [file pone.0212214.s013.docx]

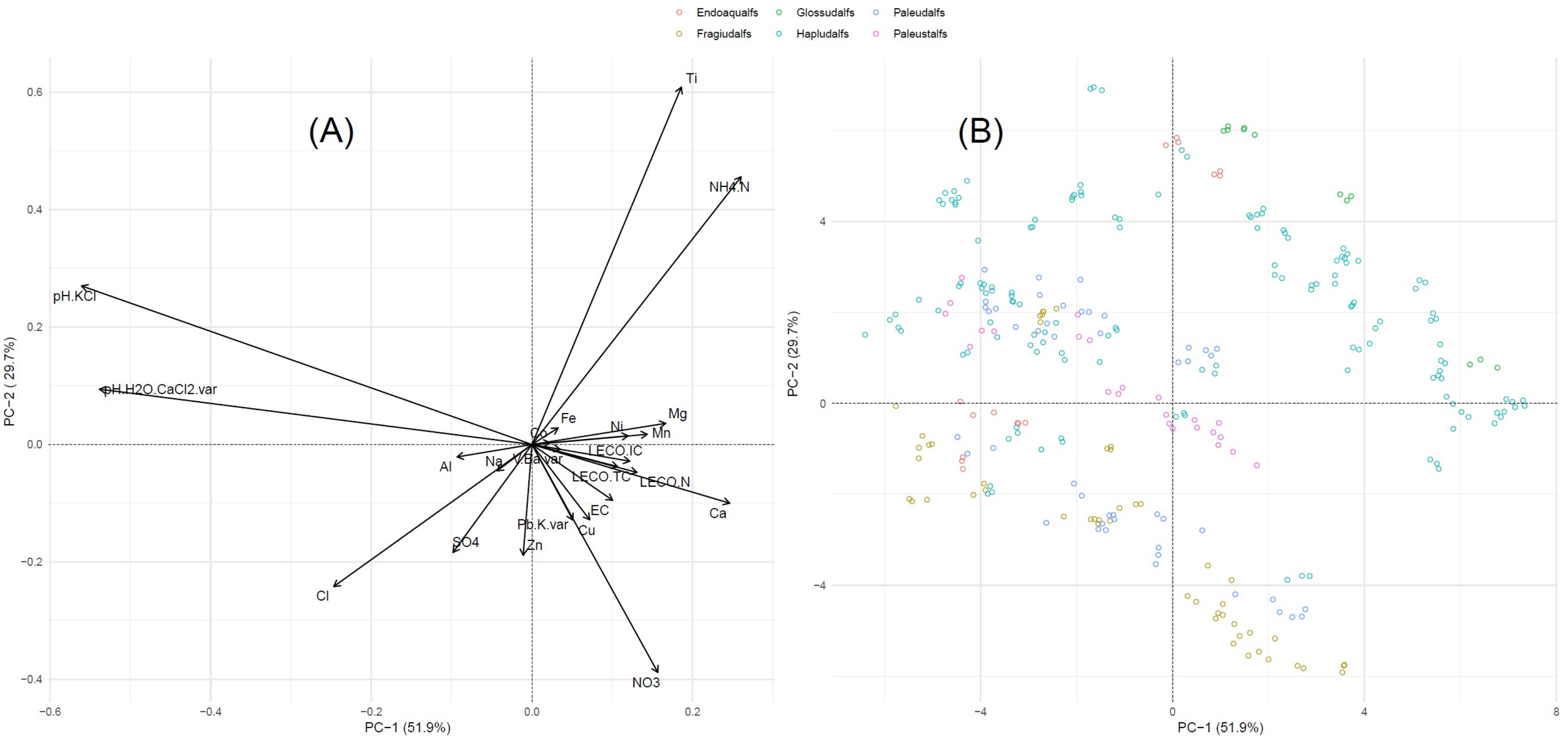

Endoaqualfs
Glossudalfs
Paleudalfs
Fragiudalfs
Hapludalfs
Paleustalfs
(A)
Ti
NH4.N
pH.KCl
pH.H2O.CaCl2.var
Fe
Co
Ni
Mg
Mn
LECO.IC
Al
Na
V.Ba.var
LECO.TC
LECO.N
Ca
EC
Pb.K.var
Cu
SO4
Zn
Cl
NO3
PC−1 (51.9%)
PC−2 (29.7%)
(B)
PC−1 (51.9%)
PC−2 (29.7%)
